# Improving Genomic Prediction of Crossbred and Purebred Dairy Cattle

**DOI:** 10.3389/fgene.2020.598580

**Published:** 2020-12-14

**Authors:** Majid Khansefid, Michael E. Goddard, Mekonnen Haile-Mariam, Kon V. Konstantinov, Chris Schrooten, Gerben de Jong, Erica G. Jewell, Erin O’Connor, Jennie E. Pryce, Hans D. Daetwyler, Iona M. MacLeod

**Affiliations:** ^1^AgriBio Centre for AgriBioscience, Agriculture Victoria Services, Bundoora, VIC, Australia; ^2^Faculty of Veterinary and Agricultural Sciences, University of Melbourne, Parkville, VIC, Australia; ^3^DataGene, Bundoora, VIC, Australia; ^4^CRV, Arnhem, Netherlands; ^5^CRV Ambreed, Hamilton, New Zealand; ^6^School of Applied Systems Biology, La Trobe University, Bundoora, VIC, Australia

**Keywords:** genomic prediction, crossbred, multi-breed, dairy cattle, GBLUP, Bayesian

## Abstract

This study assessed the accuracy and bias of genomic prediction (GP) in purebred Holstein (H) and Jersey (J) as well as crossbred (H and J) validation cows using different reference sets and prediction strategies. The reference sets were made up of different combinations of 36,695 H and J purebreds and crossbreds. Additionally, the effect of using different sets of marker genotypes on GP was studied (conventional panel: 50k, custom panel enriched with, or close to, causal mutations: XT_50k, and conventional high-density with a limited custom set: pruned HDnGBS). We also compared the use of genomic best linear unbiased prediction (GBLUP) and Bayesian (emBayesR) models, and the traits tested were milk, fat, and protein yields. On average, by including crossbred cows in the reference population, the prediction accuracies increased by 0.01–0.08 and were less biased (regression coefficient closer to 1 by 0.02–0.16), and the benefit was greater for crossbreds compared to purebreds. The accuracy of prediction increased by 0.02 using XT_50k compared to 50k genotypes without affecting the bias. Although using pruned HDnGBS instead of 50k also increased the prediction accuracy by about 0.02, it increased the bias for purebred predictions in emBayesR models. Generally, emBayesR outperformed GBLUP for prediction accuracy when using 50k or pruned HDnGBS genotypes, but the benefits diminished with XT_50k genotypes. Crossbred predictions derived from a joint pure H and J reference were similar in accuracy to crossbred predictions derived from the two separate purebred reference sets and combined proportional to breed composition. However, the latter approach was less biased by 0.13. Most interestingly, using an equalized breed reference instead of an H-dominated reference, on average, reduced the bias of prediction by 0.16–0.19 and increased the accuracy by 0.04 for crossbred and J cows, with a little change in the H accuracy. In conclusion, we observed improved genomic predictions for both crossbreds and purebreds by equalizing breed contributions in a mixed breed reference that included crossbred cows. Furthermore, we demonstrate, that compared to the conventional 50k or high-density panels, our customized set of 50k sequence markers improved or matched the prediction accuracy and reduced bias with both GBLUP and Bayesian models.

## Introduction

The interest in providing genomic predictions for crossbred dairy cows has increased especially in recent years (Harris, 2005; Sørensen et al., 2008; VanRaden et al., 2020). Crossbreeding in dairy cattle is common in New Zealand (making up almost 50% of the milking herd according to New Zealand Dairy Statistics 2018–2019)^1^, where often the aim of crossbreeding between Holstein (H) and Jersey (J) breeds is to combine the best of both breeds, and crossbred dairy bulls are commonly mated to crossbred cows (Harris, 2005; Harris and Johnson, 2010b). In addition to heterosis and breed complementarity effects, in recent years, crossbreeding is considered more as a potential approach to improve sustainability in dairy cattle breeding by reducing problems related to inbreeding and to improve fertility, survival, and other functional traits (Sørensen et al., 2008). Consequently, the number of genotyped crossbred animals is growing, and both New Zealand and United States already provide genomic evaluations for dairy crossbreds (Winkelman et al., 2015; VanRaden et al., 2020).

The establishment of a suitable reference population for crossbred predictions in dairy cattle is challenging because ideally the same reference population should be used to predict the purebreds for more than a single breed. This is because genomic evaluations for dairy cattle are typically very computationally intensive; they are undertaken at a national level for all dairy animals, involve millions of animal records from both purebred and crossbred animals, and are re-analyzed several times per year. Furthermore, the reducing cost of genotyping has resulted in very large numbers of cows being genotyped in addition to bulls because commercial farmers are interested in using genomic prediction to select female replacement animals (e.g., millions of animals in the United States; VanRaden et al., 2020). While it is possible that a combination of purebred and crossbred animals would be the ideal reference population for crossbreds, it is uncertain that this would be the optimal reference population for the purebred animals. For purebred dairy cattle, genomic prediction (GP) is often performed within a single purebred reference population because often the accuracy of predictions show high reliability, whereas the accuracy of across-breed GP is low (Kemper et al., 2015).

The accuracy of GP is highly dependent on the linkage disequilibrium (LD) between causal mutations and the dense single nucleotide polymorphism (SNP) markers spread across the genome (Meuwissen et al., 2001; Habier et al., 2007). Hence, within-breed GP in major dairy purebreds using a standard 50k chip (Illumina Bovine SNP50K) works well and has been adopted in the dairy industry of many countries (Hayes et al., 2009b). Furthermore, for within-breed GP in dairy cattle breeds, previous studies showed that there was no, or limited, gain in accuracy due to an increase in marker density (Harris and Johnson, 2010a; Su et al., 2012; VanRaden et al., 2013).

The estimated SNP effects from the reference population would be generally applicable for GP in another population if the LD between SNP and causal mutations remains the same or is very similar across the populations. However, across-breed GP, which uses the estimated SNP effects from one breed to calculate genomic estimated breeding values (GEBV) in another breed, generally shows a low accuracy. For example, using H as a reference for GP in J and *vice versa* is reported to produce a much-lower-accuracy GEBV compared to within-breed GP (Harris et al., 2008). This could be partially associated with the high conservation of LD between markers using standard 50k chip within H or J breeds, whereas to reach almost the same amount of LD across breeds would require about 300,000 SNPs (de Roos et al., 2008). Furthermore, there might be some causal mutations which do not segregate in all breeds or their allele effects differ in different breeds due to epistasis and differences in allele frequencies (Goddard et al., 2018). In across-breed GP, the increase in accuracy of GEBV using high-density (HD) genotypes (>600 k SNP) compared to 50 k SNP has also been reported to be limited (Harris et al., 2008; Erbe et al., 2012).

Combining data from different pure breeds into a single large reference population compared to within-breed GP and using HD instead of lower-density genotypes for multi-breed GP have been reported to show small gains (up to about 5%) in the accuracy of predictions (Pryce et al., 2011; Erbe et al., 2012; Hozé et al., 2014; Kemper et al., 2015; Goddard et al., 2018). Furthermore, a multi-breed reference over-dominated by one breed has recently been reported to reduce the accuracy of prediction in the breed with a minor contribution to the reference population (van den Berg et al., 2020).

Instead of increasing the overall density of SNP, an alternative approach to improve GP for both crossbred and purebred performance might be to increase the “functional density” of markers on medium-density SNP chips by enrichment with causal mutations. Then, individuals could be genotyped with a lower-priced custom medium-density SNP chip, and the GP should not suffer from an excessive number of markers for which effects should be estimated (Goddard et al., 2018). Given the paucity of functional information and millions of variants across the genome, obtaining a custom set of variants is challenging because preferably the set should be useful for predicting multiple traits. VanRaden et al. (2017) reported that using the imputed HD genotypes increased the reliabilities of GP by only 0.6 percentage points, but adding a subset of 16,648 SNP with the largest estimated effects to the 60,671 conventional SNP genotypes increased reliabilities by 2.7 percentage points. Xiang et al. (2019) proposed a comprehensive method to rank sequence variants with functional and evolutionary significance combined with their multi-trait associations across 34 important dairy traits. These authors then used this ranking together with further analyses to prioritize a custom set of medium-density markers (∼50,000) for a cost-effective SNP panel that we will refer to here as the “XT_50k chip.”

In simulation studies, it has been shown that the accuracy of GP for crossbred animals can be increased by combining pure breeds into a single reference population if the LD between markers and causal mutations is well conserved across pure breeds (Esfandyari et al., 2015b). Additionally, Esfandyari et al. (2015a) reported that using crossbreds in the reference population improved the accuracy of crossbred predictions. It is possible to account for the breed origin of alleles, but this showed no consistent advantage over a multibreed approach in real pig data, and accurately allocating a specific breed origin to alleles was an added complication (Esfandyari et al., 2015a; Sevillano et al., 2016; Vandenplas et al., 2016). An alternative and more straightforward method for GP in crossbred is to use the estimated breed proportions of each animal to calculate a weighted average of the breeding values (WA_GEBV) from two or more purebred reference populations (VanRaden et al., 2020). This approach is especially useful when few crossbred animals are available for the reference population, for example, because crossbreds are not routinely phenotyped. However, a limitation of the method is that crossbreds cannot be exploited in the reference population.

In GP, using multi-breed populations, there is evidence that Bayesian statistical methods can improve the accuracy of GEBV compared to GBLUP methods (Hayes et al., 2009a; Lund et al., 2016; van den Berg et al., 2019). In GBLUP, the prior assumption is that SNP effects are from a single normal distribution, and therefore all have small but non-zero effects (Meuwissen et al., 2001). However, Bayesian models assume that the SNP effects follow a non-normal distribution (Meuwissen et al., 2001; Habier et al., 2011) or a mixture of normal distributions with a proportion of SNP having a zero effect such as in BayesR (Erbe et al., 2012; Wang et al., 2015; MacLeod et al., 2016). Therefore, in a multi-breed reference where LD between causal mutations and markers is preserved at shorter distances, Bayesian models should be able to fine-map quantitative trait loci (QTL) more precisely and produce GEBV with high accuracy than GBLUP (Toosi et al., 2010; Goddard et al., 2018). Accordingly, MacLeod et al. (2014) found that a multi-breed reference with a Bayesian approach outperformed GBLUP for GP in animals that had low relatedness to the reference set.

We propose that a single multi-breed reference population including crossbreds, coupled with a set of markers selected to be closer to causal mutations and a Bayesian prediction model, could be beneficial for GP in crossbreds while also maintaining or improving accuracy in purebreds compared to single breed reference populations.

In the first part of this study, we assessed the accuracy and bias of GEBV for purebred and crossbred H and J cows using within-breed, across-breed, and multi-breed GP strategies. The first aim was to investigate the effect of including crossbreds in the reference population on purebred and crossbred GP. The second aim was to test three sets of markers: (a) the Illumina Bovine 50k SNP marker panel, (b) a custom set of ∼46 k markers enriched for putative causal mutations (XT_50k), and (c) the Illumina Bovine HD SNP chip augmented with approximately 1,000 custom SNP (HDnGBS). The third aim was to compare the accuracy of GP using the GBLUP or Bayesian (emBayesR) methods for all the above reference sets and marker sets.

In the second part of the study, we compared the accuracy and bias of GP using a multi-breed reference population that was either H-dominated or had balanced-breed proportions in which the potential negative effects of unequal contribution of the breeds on GP could be avoided. We also explored the benefits of including crossbred cows in the balanced-breed reference population. Similar to the first part of the study, GP was performed with three sets of markers and using GBLUP and emBayesR approaches.

## Materials and Methods

### Animals

The animals used in this study were available from CRV and consisted of 14,987 pure H (5,409 H bulls, 953 Red H bulls, and 8,625 H cows), 5,016 pure J (1,101 J bulls and 3,915 J cows), and 20,281 crossbred cows. All cows were born in New Zealand, and the bulls were from New Zealand or Netherlands. The crossbred cows were further divided to three subgroups according to the H:J breed composition as described in “Breed Allocation” (10,125 ∼75%H:25%J, 8,675 ∼50%H:50%J, and 1,481 ∼25%H:75%J).

### Reference Sets

We designed different reference sets to assess GP within breed, across breed, and for crossbreds (including or excluding crossbred cows). Furthermore, we studied the potential benefits of using a balanced-breed instead of a H-dominated reference population.

The different reference sets are shown in Table 1. Ref. 1 and Ref. 2 consisted of all pure H and all pure J animals, respectively. Ref. 3 contained all purebred (H and J), and Ref. 5 consisted of all purebred and crossbred animals. Ref. 4. and Ref. 4′ were both based on two separate single-breed reference populations (Ref. 1 and Ref. 2) but where the predictions were proportionally combined for the crossbred prediction and the single reference prediction used for the purebreds. This follows the United States dairy evaluation approach for crossbred cows (VanRaden et al., 2020). For Ref. 4, the breed proportions of validation cows were defined by using a principal component analysis (PCA) of their genomic relationship matrix (GRM) to compare and correct as needed the pedigree defined by a four-letter breed group based on paternal and maternal grandparents. For Ref. 4′, Admixture software (Alexander et al., 2009) was used to define continuous breed proportions with the assumption that there were only two breeds in the population (*k* = 2). Ref. 6, Ref. 7, and Ref. 8 had balanced-breed proportions, and all had the same set of 2,202 bulls (1,101 H and 1,101 J) but differed in the cows added in. Ref. 6 included equal numbers of pure H and J cows, while Ref. 7 contained equal numbers of only crossbred cows. Ref. 6 and Ref. 7 also contained the same number of animals. Finally, Ref. 8 included both the purebred cows from Ref. 6 and the crossbred cows from Ref. 7, and this was close in the number of animals to Ref. 3 (H-dominated). The subsets of animals used in these balanced-breed reference sets were sampled randomly from the full reference to avoid changes in the average relationships between the reference animals and the validation animals. Otherwise, any non-random sampling from the full reference could result in the subset being more/less closely related to the validation set, and this would confound the results of GP when compared with the full reference. To help better differentiate between GP approaches, the reference animals and validation sets were intentionally allocated to minimize very highly related individuals between these groups.

**TABLE 1:** Number of purebred and crossbred animals in different reference sets.

		**Purebred bull**			**Purebred cow**		**Crossbred cow**		
**Reference acronym^1^**	**Total number**	**H**	**Red-H**	**J**	**H**	**J**	**75%H:25%J**	**50%H:50%J**	**25%H:75%J**
Ref. 1	13,985	4,407	953	–	8,625	–	–	–	–
Ref. 2	4,484	–	–	1,101	–	3,383	–	–	–
Ref. 3	18,469	4,407	953	1,101	8,625	3,383	–	–	–
Ref. 4	18,469	4,407	953	1,101	8,625	3,383	–	–	–
Ref. 5	36,695	4,407	953	1,101	8,625	3,383	9,262	7,807	1,157
Ref. 6	8,968	1,101	–	1,101	3,383	3,383	–	–	–
Ref. 7	8,968	1,101	–	1,101	–	–	1,157	4,452	1,157
Ref. 8	15,734	1,101	–	1,101	3,383	3,383	1,157	4,452	1,157

*^1^The multi-breed Refs. 6–8 represent balanced-breed sets while Refs. 3–5 are Holstein-dominated sets.*

### Validation Sets

The same validation cows were used in all comparisons. The cows in the validation sets were selected to avoid high relationships with animals in the reference set that included all pure and crossbred animals (i.e., Ref. 3), so there were no sires, full-sib brothers and sisters, and maternal half-sib sisters and half-sib brothers of validation cows in the reference sets. The number of paternal half-sib sisters were restricted to be as low as possible. The cow validation set consisted of 3,589 cows divided into five breed groups as described in “Breed Allocation” (H, ∼75%H:25%J, ∼%50 H:50%J, ∼%25 H:75%J, and J). Table 2 shows the number of cows in each validation breed group and the number of their sires, in addition to the average, standard deviation, and median number of paternal half-sib sisters of validation cows across different reference sets.

**TABLE 2:** Description of the validation cow sets. Included is the number of cows in each breed group, number of sires that they represented, and average ± standard deviation (median) of the number of paternal half-sib sisters (HSS) of validation cows in the different reference sets (details of reference sets in Table 1).

	**H**	**75%H:25%J**	**50%H:50%J**	**25%H:75%J**	**J**	**Total**
Number of cows	1,002	863	868	324	532	3,589
Number of sires	314	381	355	128	136	951
Ref. 1 HSS	1.37 ± 1.83 (0)	0.83 ± 1.47 (0)	0.42 ± 0.9 (0)	1.21 ± 1.21 (1)	1.16 ± 1.18 (1)	0.96 ± 1.46 (0)
Ref. 2 HSS	0.04 ± 0.36 (0)	0.18 ± 0.56 (0)	0.96 ± 2.57 (0)	6.51 ± 8.31 (3)	9.85 ± 10.97 (6)	2.33 ± 6.23 (0)
Refs. 3 and 4 HSS	1.41 ± 1.92 (0)	1 ± 1.63 (0)	1.38 ± 2.96 (0)	7.72 ± 9.09 (5)	11.02 ± 12.07 (8)	3.3 ± 6.82 (0)
Ref. 5 HSS	6.55 ± 8.58 (2)	10.34 ± 13.31 (3)	10.07 ± 13.63 (3)	76.4 ± 83.63 (37)	55.93 ± 62.69 (29)	21.94 ± 43.48 (4)
Ref. 6 HSS	0.79 ± 1.36 (0)	0.59 ± 1.23 (0)	1.12 ± 2.71 (0)	7.31 ± 8.78 (3)	10.41 ± 11.36 (7)	2.84 ± 6.49 (0)
Ref. 7 HSS	1 ± 1.8 (0)	3.53 ± 5.41 (1)	4.6 ± 6.57 (1)	36.01 ± 39.3 (18)	26.43 ± 28.97 (13)	9.41 ± 20.55 (1)
Ref. 8 HSS	1.78 ± 2.66 (1)	4.12 ± 5.87 (1)	5.72 ± 8.29 (1)	43.32 ± 45.69 (23)	36.84 ± 38.55 (23)	12.24 ± 25.85 (1)

### Phenotypes

The phenotypes of milk traits (milk, fat, and protein yields) for CRV bulls were de-regressed proofs (DRP) on the Australian scale, derived from international within-breed MACE (2018) breeding values (Liu, 2009). The phenotypes for the cows were also DRP equivalents calculated by DataGene in 2018 using test day records with random regression models and correcting for the fixed effects (herd, year, season, lactation) following the approach used for the official Australian dairy cattle evaluations^2^. It was convenient to use all data processed on the Australia scale because they were available as part of another research project described in Haile-Mariam et al. (2020) combining Australian and New Zealand data.

### Genotypes

Three different sets of markers were evaluated for GP:

(1) conventional Illumina Bovine50k SNP panel with 40,850 SNP after quality control and that overlapped the Illumina BovineHD panel;
(2) Illumina BovineHD 800k SNP panel with an additional custom set of about 1,000 SNP (HDnGBS). This set was then pruned for strong LD where one of each pair of SNP in LD *r*^2^ > 0.95 was pruned out using PLINK (Purcell et al., 2007). This reduced the number of SNP from 633,375 to 316,396 (pruned HDnGBS), making genomic prediction analysis more computationally efficient. We tested the accuracy of the full panel *versus* the pruned panel in several analyses and found no significant difference between the full and the reduced marker sets, so we presented only the GP with pruned HDnGBS genotypes in this paper; and
(3) customized set of 46,516 SNP (XT_50k) which were selected from whole genome sequence according to multiple criteria to be closer to or potentially the causal mutations for 34 economically important traits in dairy cattle (Xiang et al., 2019, 2020).

Most of the genotypes in our study were first imputed from lower-density chips (approximately 8,000 SNP overlapping the 50k panel) up to standard 50k and then imputed from 50k to HD using FImpute (Sargolzaei et al., 2014). Pedigree information was not used for imputation. The HD SNP set was imputed to the whole genome sequence using Minimac3 (Das et al., 2016) having pre-phased the data with Eagle2 (Loh et al., 2016). Run6 version of the 1,000-bull genome (Daetwyler et al., 2014; Bouwman et al., 2018) was used as the sequence imputation reference, and this was also pre-phased with Eagle2 prior to imputation of the HD genotypes. The custom set of ∼1,000 SNP and XT_50k SNP was extracted from imputed whole genome sequence. The LD pruning process for the HDnGBS set was done with consideration of preferentially removing SNP tagging the custom set of ∼1,000 SNP. Finally, before performing GP, SNP with minor allele frequency less than 0.002 were removed.

### Breed Allocation

The bulls in our study were purebred by pedigree and allocated to the H or J breed groups accordingly. However, the cows were allocated to purebred and crossbred (sub)groups according to their pedigree information and the first principal component (PC) calculated from the GRM using GCTA (Yang et al., 2011) on a core set of 8,185 autosomal low-density SNP that had been genotyped in all animals. This was done because not all cows had full breed information and some had incorrect breed codes. The bull and cow four-letter breed code that depicts the maternal and paternal grandparent breed based on pedigree was used to set the first PC boundaries of each group, and the PCA was used to correct breed codes that appeared incorrect or were incomplete. The prediction of breed proportion was also performed in Admixture software (Alexander et al., 2009) using the same SNP set and including the New Zealand purebred bulls and cows. The number of ancestral populations (*k*) in Admixture was set to equal the expected number of breeds (H and J: i.e., *k* = 2).

### Genomic Prediction

We performed GP with two statistical methods, Genomic Best Linear Unbiased Prediction (GBLUP) (Meuwissen et al., 2001) and emBayesR (Wang et al., 2015).

#### GBLUP

The GEBV for the animals were calculated using MTG2 (Lee and van der Werf, 2016) and by fitting the model shown in Equation 1 for each of the reference sets and each of the milk traits (milk, fat, and protein yields). Furthermore, GEBV were calculated using three different marker sets (50k, XT_50k, or pruned HDnGBS genotypes) to construct the GRM in the model (Yang et al., 2010).

(1) y=Xb+Zu+e

where **y** is a vector of DRP for the milk traits (milk, fat, or protein yields) of the animals in the reference, **X** is a design matrix allocating DRP to fixed effects, **b** is the vector of fixed effects (mean, sex, and breed group), and **Z** is a design matrix allocating DRP to GEBV in vector **u**. The variance of the breeding values is calculated as Var(**u**) = **G**σ^2^_*g*_, where σ^2^_*g*_ is the additive genetic variance, **G** is the GRM constructed from genotypes of the animals in the reference and validation sets, and **e** ∼ (0, **E**σ^2^_*e*_) is a vector of random residual effects in which σ^2^_*e*_ is the error variance and **E** is a diagonal matrix as diag(**E**)_*i*_ = 1/*w*_*i*_, where *w*_*i*_ is the weighting coefficient for the *i*th animal. Weighting coefficients were calculated differently for cows and bulls using Eqs. 2 and 3, respectively (Garrick et al., 2009).

(2) $$w_{C\,o\,w}=\frac{1-h^{2}}{{\mathrm{ch}}^{2}+\frac{1+\left(n-1\right)\,t}{n}-h^{2}}$$

(3) $$w_{b\,u\,l\,l}=\frac{1-h^{2}}{{\mathrm{ch}}^{2}+\frac{\left(4-h^{2}\right)}{p}}$$

where *h*^2^ is heritability (=0.33), *t* is repeatability (=0.56), *c* is the proportion of variance not explained by markers (=0.2), *n* is the number of records for each cow, and *p* is the number of daughters for each bull.

#### emBayesR

Genomic estimated breeding values for the animals were also calculated with emBayesR method (Wang et al., 2015) using an in-house software and fitting the model shown in Equation 4. Benefiting from an approximate EM algorithm in the initial phase, emBayesR is a faster approach for GP compared to fully dependent Markov chain Monte Carlo (MCMC) algorithm in BayesR (Erbe et al., 2012) while still sampling the SNP effects from a mixture of normal distributions.

(4) y=Xb+Wv+e

where **y**, **X**, **b**, and **e** are as described in Eq. 1, **v** is the vector of estimated SNP effects, and **W** is a design matrix of SNP genotypes that were standardized to have a variance of 1. The proportion (and the additive genetic variance) of the SNP effects sampled from four normal distributions were set to 0.94 (0), 0.049 (0.0001), 0.01 (0.001), and 0.001 (0.01). Thus, for example, each SNP had 94% prior chance to have 0 contribution in explaining the genetic variance of the trait. The number of iterations in the emBayesR analyses was adapted to achieve consistent results across the five chains, requiring 1,500 to 2,200 EM iterations with the convergence parameter set as 1 × 10^–7^ and 5,000 to 15,000 BayesR iterations. Finally, the results were averaged across the five MCMC chains.

#### Validation

In all reference sets, other than Ref. 4 and Ref. 4′, the GEBV were calculated for the validation cows similar to reference animals but masking their phenotypes in Eqs. 1 and 4. In Ref. 4 and Ref. 4′, the breed proportion was used to calculate a weighted average of the two GEBV (WA_GEBV) calculated from purebred H and J reference sets (VanRaden et al., 2020). In Ref. 4, the GEBV for each animal in the validation set was calculated by multiplying their GEBV from both Ref. 1 (H only) and Ref. 2 (J only) by the proportion of H and J breeds estimated according to the approximate breed groups allocated through PCA and pedigree information. In Ref. 4′, the GEBV were calculated as for Ref. 4, but using the exact breed proportions estimated from Admixture software.

The accuracy and bias of GP for each of the five validation breed groups were calculated separately. The accuracy was the Pearson’s correlation coefficient between GEBV and DRP, and the bias of GP was assessed by calculating the regression coefficient of DRP on GEBV, so the GP was least biased when the regression coefficient showed the least deviation from one.

## Results

### Breed Group Allocation

**FIGURE 1:**
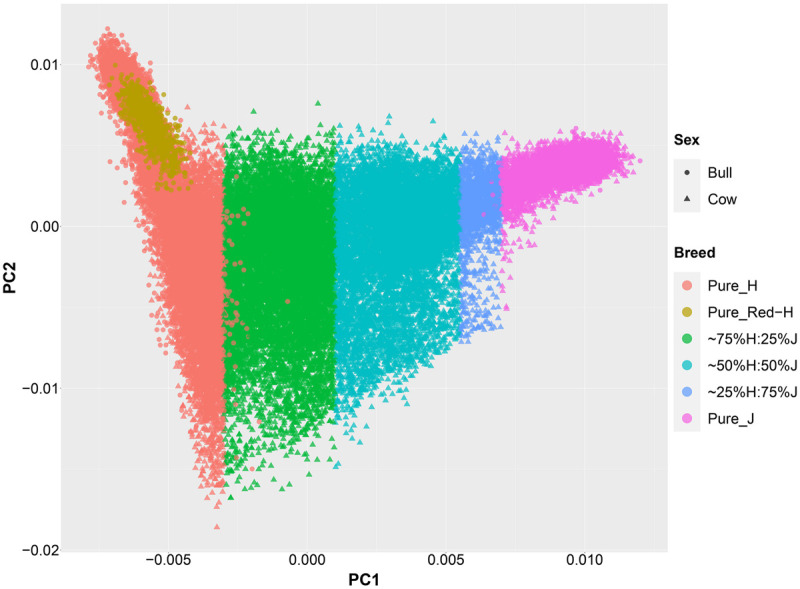
The second principal component (PC) is plotted against the first PC of the genomic relationship matrix constructed using a low-density set of genotypes of all purebred and crossbred animals in this study.

**FIGURE 2:**
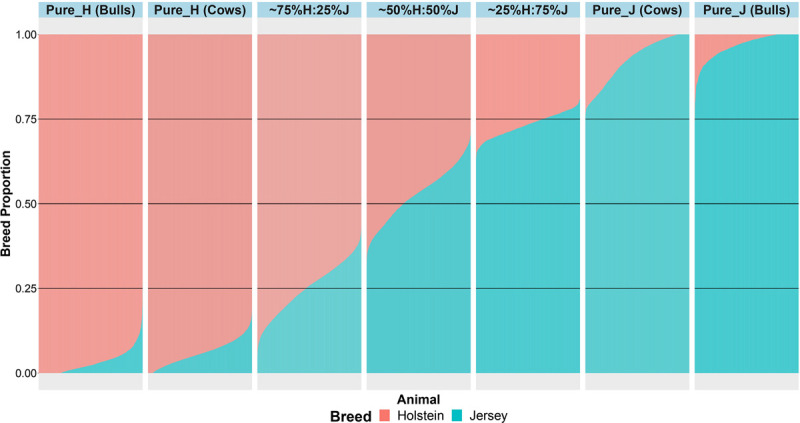
The admixture breed proportions estimated with Admixture software where each horizontal line represents the breed proportion of each animal.

An important aspect of this study was to ensure that the cows were correctly allocated to breed groups because crossbred cows in New Zealand are sometimes inter-crossed for several generations through the use of crossbred bulls, and some cows had incomplete or incorrect pedigree breed definitions. A combination of pedigree breed codes and a PCA of the GRM were used to allocate cows to five breed categories (H, 75%H:25%J, 50%H:50%J, 75%J:25%H, and J; Figure 1). This breed group allocation was then also evaluated with Admixture software as shown in Figure 2. Generally, the exact breed proportions predicted in Admixture matched well with the approximated breed proportion using PCA and pedigree information.

### Reference Populations: Refs. 1–5

**FIGURE 3:**
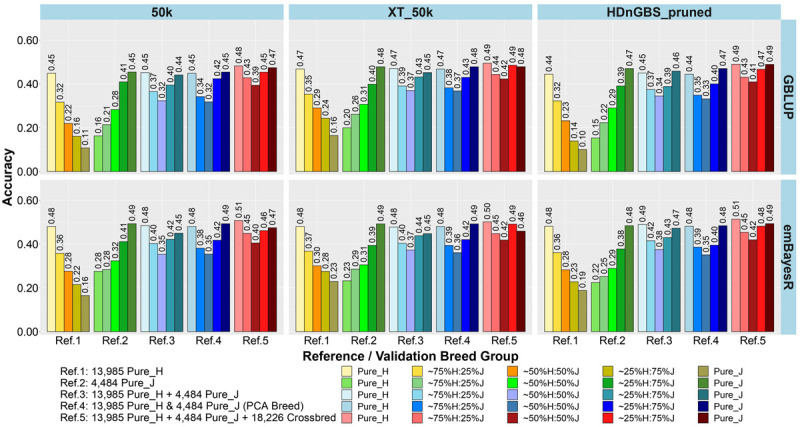
Accuracy of genomic predictions in five validation sets using different reference populations (Refs. 1–5: details in Table 1). The results are averaged across milk, fat, and protein yields.

**FIGURE 4:**
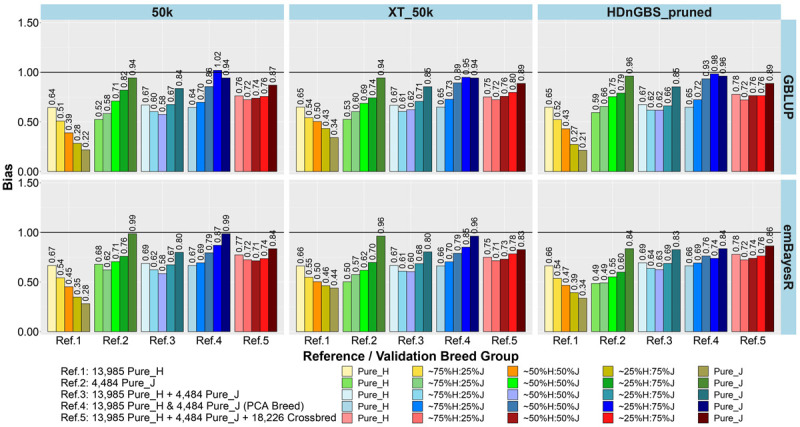
Bias of genomic predictions in five validation sets using different reference populations (Refs. 1–5: details in Table 1). The results are averaged across milk, fat, and protein yields.

In the first part of our study, we compared the accuracy and bias of GP using reference sets (Table 1) that were either single breed (Refs. 1 and 2), a mix of purebreds (Refs. 3 and 4), or a mix of purebreds and crossbreds (Ref. 5). The main focus of testing different reference sets was to determine if there were reference sets that work equally well for crossbred and purebred GP. The results for the accuracy and bias of GP in the five breed group validation sets are shown as an average across three milk traits (milk, fat, and protein yields) in Figures 3, 4, respectively, because the results showed consistent trends across these traits for all comparisons. However, the individual trait results for different GP scenarios are provided in Supplementary Figures 1, 2.

The comparison between different reference sets showed that across-breed GP (i.e., predicting H from the reference consisting of only purebred J and *vice versa*) had the lowest accuracy and largest bias. As expected, the within-breed GP performed well (i.e., predicting H and J from reference sets consisting of only purebred H and purebred J, respectively). The crossbred validation group with breed composition closest to the purebred reference set had the second best accuracy of GP in Refs. 1. and 2, while a steep decline was seen in the other crossbred groups using these single-breed reference sets.

Combining purebred H (Ref. 1) and J (Ref. 2) animals into a single reference (Ref. 3) resulted in an average increase in the accuracy by 0.09 and a reduction of bias by 0.08 for crossbreds compared to single breed references. In Ref. 3 compared to Ref. 1, on average, ∼25%H:75%J cows achieved a maximum gain in accuracy (0.21) and reduction in bias (0.32). However, for purebred J cows, the H-dominated Ref. 3 compared to Ref. 2 reduced their accuracy by about 0.03 and considerably increased the bias by 0.11.

In Ref. 4, we proportionally combined GEBV derived from the H and J single-breed reference sets (Refs. 1 and 2) according to the approximated PCA breed proportions for each validation set. Although this method did not improve the accuracy of predictions compared to Ref. 3, it did on average reduce the bias by 0.18. The average reduction in bias was highest in ∼50%H:50%J (0.23), followed by ∼25%H:75%J (0.21) and ∼75%H:25%J (0.09). We also tested substituting these approximate breed proportions with the exact Admixture breed composition for each cow to calculate their GEBV (Ref. 4’). The accuracy and bias were similar to Ref. 4, and these can be seen in Supplementary Figure 1 (labeled Ref. 4′).

In Ref. 5, generated by adding crossbred cows to Ref. 3 (H and J), the accuracy increased by between 0.03 to 0.08 in crossbreds compared to Ref. 3 and Ref. 4. For Ref. 5, in comparison to Ref. 3, there was an average reduction in bias for all validation breed groups that was highest in ∼50%H:50%J (0.14), followed by ∼75%H:25%J (0.11) and ∼25%H:75%J (0.09), compared to purebred cows with a reduction in bias of GEBV (0.06). However, in comparing Ref. 5 to Ref. 4, there was only a reduction in bias for the pure H and the ∼75%H:25%J, while on average the bias increased for the ∼50%H:50%J, 25%H:75%J, and pure J breed groups.

### Genotypes: Marker Sets 50k, XT_50k, and Pruned HDnGBS (Refs. 1–5)

For single-breed references, comparing three different sets of markers (Figures 3, 4) showed that using XT_50k or pruned HDnGBS instead of 50k increased the accuracy of GP for within-breed prediction (H and J) by about 0.02. In Ref. 1, using XT_50k (and pruned HDnGBS) instead of 50k consistently improved the accuracy of GP for crossbred cows by, on average, 0.05 (and 0.04) and also reduced bias by about 0.08 (and 0.06). In reference sets 3, 4, and 5, there was also a small but consistent advantage in the crossbred GBLUP accuracy for the XT_50k set over the 50k and pruned HDnGBS, but there were no consistent differences in the accuracies using emBayesR. In reference sets 3, 4, and 5, there was no consistent trend for bias across the three marker sets.

### Methods: GBLUP Versus emBayesR (Refs. 1–5)

Comparing the two different statistical methods for GP (Figures 3, 4), it was shown that the emBayesR method gave a consistent increase in accuracy compared to GBLUP for crossbred and purebred prediction using single-breed reference sets (Refs. 1 and 2). On average, there was also a small but consistent advantage in accuracy for emBayesR *versus* GBLUP in Refs. 3, 4, and 5 for 50k and pruned HDnGBS marker sets. However, the benefits of emBayesR over GBLUP in accuracy diminished with the use of the custom XT_50k marker set. The differences in bias between emBayesR and GBLUP were less consistent: for example, in Ref. 3, emBayesR reduced the bias of GP in crossbred cows by about 0.03 compared to GBLUP, but the bias was similar for both methods in Ref. 5.

### Equalizing Breed Proportions in Reference Sets

**FIGURE 5:**
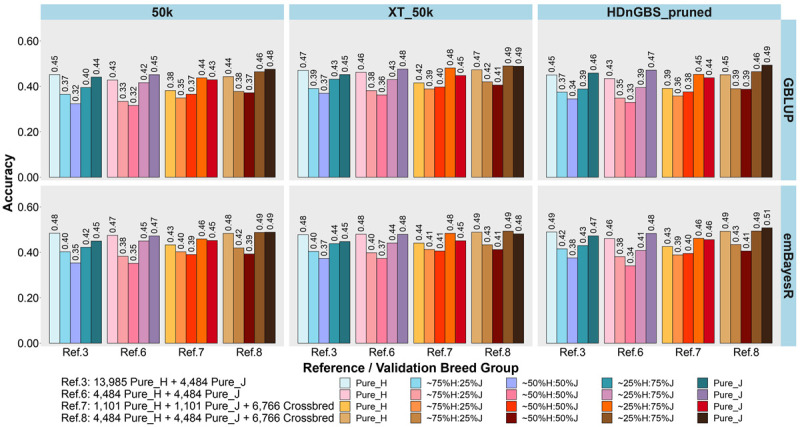
Accuracy of genomic predictions in five validation sets using different reference populations (Ref. 3 and Refs. 6–8: details in Table 1). The results are averaged across milk, fat, and protein yields. Ref. 3 is Holstein-dominated, while Refs. 6–8 have balanced-breed proportions.

**FIGURE 6:**
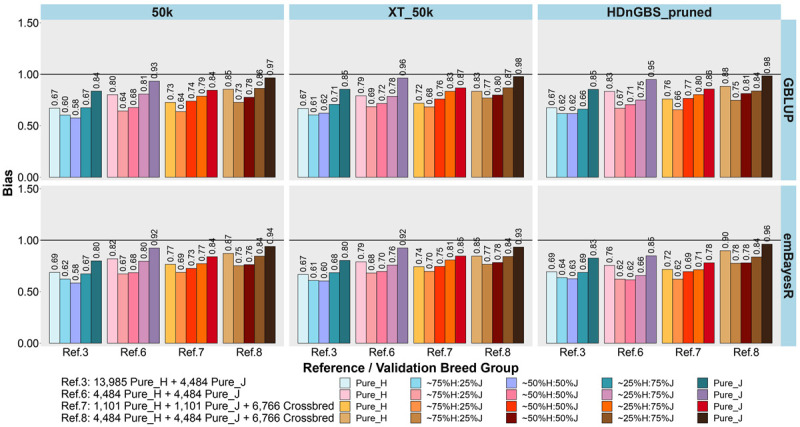
Bias of genomic predictions in five validation sets using different reference populations (Ref. 3 and Refs. 6–8: details in Table 1). The results are averaged across milk, fat, and protein yields. Ref. 3 is Holstein-dominated, while Refs. 6–8 have balanced-breed proportions.

In the second part of our study, we compared the accuracy and bias of GP in Ref. 3 (mixed H and J purebreds and dominated by H) *versus* three additional reference sets, where breed proportion was equalized (Refs. 6, 7, and 8: Table 1) in Figures 5, 6. Refs. 6, 7, and 8 all included the same ∼2,200 H and J bulls but differed in cow composition: purebreds (Ref. 6), crossbreds (Ref. 7), or pure and crossbreds (Ref. 8).

First, comparing H-dominated Ref. 3 that had ∼18,500 purebred animals *versus* Ref. 6 that had only ∼9,000 purebreds balanced across H and J (all J animals from Ref. 3 but the H set randomly reduced from ∼14,000 to ∼4,500 animals), on average, the bias was considerably reduced for all validation sets in Ref. 6, with the most impact in purebreds (reducing by 0.14 for H, 0.10 for J, 0.06 for 75%H:25%J, 0.09 for 50%H:50%J, and 0.10 for 25%H:75%J). The accuracies were similar to Ref. 3, but there was a consistent trend for the H accuracy to fall in Ref. 6 by 0.01 to 0.02 and J to increase by 0.01 to 0.02. Therefore, GP in the J breed benefited from simply removing a large proportion of H to achieve similar breed proportions in Ref. 6, resulting in both bias and accuracy being restored to similar levels as using purebred J reference (Ref. 2).

Ref. 7 had the same number of animals as Ref. 6, but crossbred cows replaced purebred cows. This resulted in a consistent average increase in the accuracy of GP for the crossbred validation sets compared to Ref. 6. and Ref. 3. However, the accuracy for the H and J purebreds consistently reduced. Ref. 8 included all the cows from Ref. 6 and 7 (pure and crosses) with ∼15,700 animals, and this restored the purebred accuracies to either the same (H) or higher (J) than Ref. 3 and Ref. 6. For the all the crossbred validation sets, accuracy was consistently increased in Ref. 8 compared to Refs. 3 and 6. There was a dramatic reduction in bias for Ref. 8 (balanced-breed) compared to the H-dominated Ref. 3 for all five validation sets: on average, the reduction of bias was 0.19 for H, 0.13 for J, and 0.16 for the crossbred validation sets. Overall, the bias was always highest in the H-dominated Ref. 3 compared to Refs. 6, 7, and 8.

**FIGURE 7:**
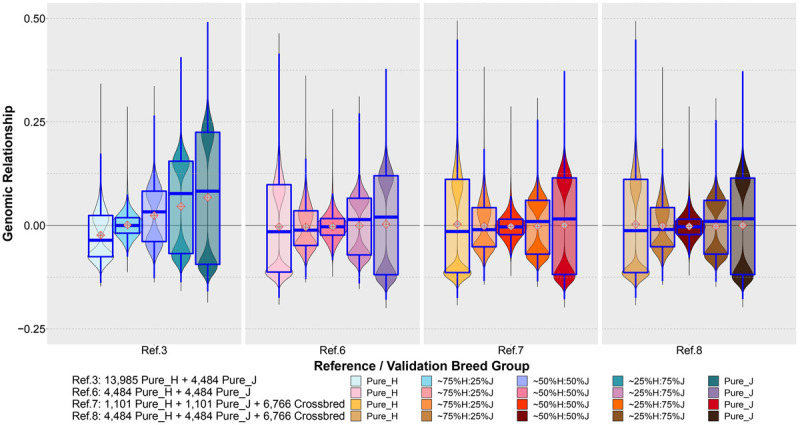
The distribution of genomic relationships estimated between a common set of bulls in Ref. 3 and Refs. 6–8 (1,101 H and 1,101 J) and the cows in the five validation breed groups. The genomic relationships were estimated using the XT_50k genotypes.

Figure 7 shows the distribution of the estimated genomic relationships between a set of purebred bulls common to Refs. 3, 6, 7 and 8 (1,101 H and 1,101 J) and the cows in the five validation breed groups. The genomic relationships displayed between these common sets of reference bulls and validation cows were estimated separately for each reference set and validation animals using the XT_50k genotypes. It can be seen that the genomic relationships had a very different distribution when estimated in the reference population that was dominated by purebred H (Ref. 3) compared to the equalized breed sets in Refs. 6, 7, and 8.

## Discussion

This study used approximately 18,500 purebred and 18,200 crossbred dairy animals to comprehensively test a range of strategies to jointly optimize the accuracy of GP for crossbreds and purebreds. A novel strategy was tested in which breed proportions were balanced in a mixed-breed reference population with the inclusion of a large number of crossbred cows and also using a custom SNP chip enriched with sequence variants. In many dairy industries, one breed dominates, while other breeds and crosses are important but have substantially lower numbers genotyped. While this study focused on GP in H and J breeds, the results are likely to be equally relevant to GP in other breeds and other livestock groups, such as beef cattle and sheep, particularly where one breed is more dominantly used compared to other breeds.

As expected, our single purebred references (Refs. 1 and 2) were suitable only for within-breed GP but provided a baseline for comparing accuracy and bias for other reference sets. These single-breed marker effects were also used to compute a WA_GEBV for crossbreds (VanRaden et al., 2020). VanRaden et al. (2020) reported the successful use of breed representation of up to five dairy breeds estimated with Findhap (VanRaden and Cooper, 2015) for the WA_GEBV. We had crossbreds of just two breeds and found a similar accuracy of WA_GEBV using either approximate breed proportions from PCA or the breed proportions estimated using Admixture software (Refs. 4 and 4′), implying that either approach was valid. However, for crossbreds of more than two breeds, it would be more practical to use software such as Admixture or Findhap to predict breed proportion. Similar to VanRaden et al. (2020), we found that the WA_GEBV approach was competitive (Ref. 4) if no crossbred phenotypes were available because it increased the accuracy compared to a single breed, reduced the bias for crossbreds compared to a multi-breed reference, and maintained purebred accuracies. Combining the purebreds into a single multi-breed reference caused our J accuracy to drop, implying a negative impact from the multi-breed reference being dominated by H. A similar finding has been reported for the accuracy of GP of Australian Red breed by van den Berg et al. (2020) when using a mixed breed reference strongly dominated by H. Previous simulation and plant studies also showed that increasing the size of the reference population by including individuals not closely related to the validation set could reduce the accuracy of GP (Neyhart et al., 2017; Mangin et al., 2019).

Although the WA_GEBV approach offers analytical simplicity, unfortunately, it does not exploit crossbred data where available. Previous studies have reported the significance of including crossbred animals in the reference for better GP (Esfandyari et al., 2015a; van Grevenhof and van der Werf, 2015). We also found that combining all the purebred and crossbred animals (Ref. 5) could improve accuracy compared to the WA_GEBV (Ref. 4), but still this incurred an increase in bias of predictions for crossbreds. We believe this is in a large part due to the domination of H breed in the reference (both purebreds and crossbreds) because the bias reduced considerably by balancing the proportion of H and J in Ref. 6 compared to the H-dominated Ref. 3. Furthermore, the genomic relationship between the common reference bulls and validation cows (Figure 7) shows very different distributions of relationships estimated in the H-dominated Ref. 3 compared to the balanced-breed reference sets 6, 7, and 8, probably due to differences in allele frequencies between the breeds. Excluding a large proportion of H and 75%H:25%J from the reference to equalize breed proportions, Ref. 6 did reduce the prediction accuracy in H and 75%H:25%J when compared to the full Ref. 8 (mixed breed and cross set). Notably, however, the accuracy for the H validation was still equal to that achieved with the purebred-H-only reference set (Ref. 1), and bias was less in Ref. 6 vs. Ref. 8. This suggests that a reference set with more balanced-breed proportions and including crossbreds may provide a practical compromise for genomic prediction for both purebreds and crosses. However, in specific cases, this will also depend on the numbers available for the minor breed because if too many H need to be removed, then the accuracy of prediction for the H will drop below that achieved from a single-breed H reference set (Ref. 7 vs. Ref. 8). In this case, for purebred H predictions, the alternative would be to combine the H purebreds with their closest crosses because our results demonstrated that the inclusion of crossbreds in the reference set improved the prediction accuracy in the purebreds (Ref. 8 vs. Ref. 6). Altogether the results highlight the importance of trying to ensure that genotyped reference sets are developed to include as many as possible of the minor breeds and their crosses to encourage genetic diversity and progress across all breed groups. While we demonstrated improved GP from the balanced-breed set that included pure and crossbred cows (Ref. 8), it is possible that this could be further improved by adding some more H and H-cross animals from the full set, provided that they are chosen to be the most closely related to the validation animals. For example, van den Berg et al. (2020) reported that combining a limited number of H closely related to Australian Red in a H-dominated multi-breed reference was the best strategy to improve the GP in Australian Red. However, in the context of trying to simultaneously improve the accuracy of both purebred and crossbred groups, this may not be straightforward. A method proposed by Harris and Johnson (2010b) within the GBLUP framework to account for differences in allele frequency between H, J, and crossbreds could be tested in future work to determine if this mitigates the H domination effect.

In our study, while the accuracy of GP for crossbred cows improved considerably with the balanced multibreed reference that included crossbreds (Ref. 8), the accuracy for crossbreds was still often lower than the accuracy of predicting purebreds. However, this could be due in part to the lower accuracy of DRP in crossbred validation cows compared to that in purebred cows and the lower relatedness, on average, of crossbred validation cows compared to purebred bulls (Figure 7). The only exception was the ∼25%H:75%J validation set that met the expected level of accuracy relative to the purebreds (Ref. 8). This may be because, on average, this set shared more half-sib sisters with Ref. 8 compared to the other two crossbred validation sets (Table 2). Therefore, in an industry setting, the accuracy of crossbreds may be found to be close to the average of parental breeds if there is high relatedness between crossbred cows in the reference population with those in the new test sets. It is also likely that the inclusion of crossbred bulls in the reference would increase the accuracy of GP in crossbred cows because crossbred bulls in New Zealand are mainly used for mating with crossbred cows (New Zealand Dairy Statistics 2018–2019)^3^.

Another model that has been tested for genomic prediction of multiple breeds and crosses is the multi-trait model, where the same trait is fitted as a correlated trait. However, this multi-trait approach for GP in dairy cattle showed no consistent improvement over a single-trait model (Olson et al., 2012; Haile-Mariam et al., 2019; van den Berg et al., 2020). Given that the correlation between DRP for milk traits for the same animals in our study was previously reported to be high (Haile-Mariam et al., 2019) and given that dairy cattle purebred and crossbred cows are raised under the same condition and even in the same herds, a multi-trait approach was not expected to improve the accuracy of GP.

Our custom panel, XT_50k, included ∼35,000 variants (out of 46,516) that were close to or included causal mutations for a range of 34 dairy traits (Xiang et al., 2019). This means that it is different to most custom panels in that the majority of SNP were selected as more highly predictive rather than the majority being random variants enriched with a smaller selected set. It is useful to evaluate the accuracy of GP in validation sets that are more distantly related to see if the LD phase between markers and QTL is preserved more strongly. Therefore, it is interesting to note that, for the pure H reference (Ref. 1), the XT_50k genotypes maintained a considerably higher accuracy in the more distantly related validation sets compared to the 50k. In fact, for the most distantly related validation sets in Ref. 1, the XT_50k accuracy even exceeded the high-density panel (HDnGBS) and, as expected, the emBayesR approach showed a higher accuracy than GBLUP. The reason for this is likely because the GBLUP model assumes an infinitesimal model where all markers have a small effect, while the emBayesR model assumes that a large proportion of the markers have no effect and also allows for a more complex genetic architecture by modeling a mixture of normal distributions, which better accommodates estimating large effect mutations such as the DGAT1 mutation for milk traits (Grisart et al., 2004).

The extra value of the XT_50k was less clear in the pure J reference (Ref. 2), which is possibly a reflection of the variant discovery work to select markers for the XT_50k being undertaken in a H-dominated set of animals (Xiang et al., 2019). However, it could also be partly influenced by the fact that the J reference set was less powerful than the H reference that was three times larger. The average improvement here of up to 6% from the XT_50k *versus* the 50k set is in line with other studies. For example, VanRaden et al. (2017) reported that adding 16,648 SNP to a 60k panel increased the reliabilities of within-breed GP when compared to HD genotypes. Brøndum et al. (2015) reported that adding 1,623 sequence variants identified by genome-wide association study from multiple breeds to a custom chip increased the reliabilites by up to 5 percentage points for production traits in French H.

It was also interesting that, while emBayesR mostly outperformed GBLUP, in our study, both approaches performed equally well for the XT_50k set with multi-breed references. Some previous studies showed that the accuracy of GBLUP models was more competitive with Bayesian models when selected QTL markers were modeled by fitting a separate GRM to that of the random markers to allow their effects to be sampled from a normal distribution with a higher variance (Khansefid et al., 2014; Brøndum et al., 2015; Moghaddar et al., 2018). It is possible that, in our study, GBLUP showed competitive accuracies to emBayesR without fitting the selected variants as a separate component because around 80% of the variants in the XT_50k set were selected as QTL markers, with only approximately 8,000 that were random markers. This makes the XT_50k custom panel quite different to those previously reported where the proportion of QTL markers was much lower than the remaining random marker set.

## Conclusion

Our study compared different reference populations, SNP marker sets, and statistical approaches (GBLUP and emBayesR) for GP in purebred and crossbred H and J cows. Generally, we found that a H-dominated reference had a negative effect on GP of J and crossbreds. Balancing the breed proportions in the reference set achieved a comparable accuracy to a H-dominated reference but a consistently reduced bias for both crosses and purebreds. Inclusion of crossbred cows in the reference population improved the accuracy especially for crossbreds. Using a custom marker panel (XT_50k) instead of standard 50k or pruned HD panels further improved the prediction accuracy and reduced the bias. Remarkably, the advantage of emBayesR over GBLUP was very limited when XT_50k genotypes were used in GP, indicating the benefits of using a selected set of markers. In conclusion, to improve crossbred GP, we recommend a balanced-breed reference containing crossbred animals and using a set of SNP close to QTL and enriched for causal mutations. Our results indicate that this may also be a competitive reference for GP in purebreds, particularly for the less numerous breeds. We also recommend further research to find an optimized method of selecting a subset of the dominant breed for a balanced reference or other corrective algorithms to mitigate the major breed domination effect on the accuracy and bias of GP in pure and crossbred cattle.

## Data Availability Statement

The data analyzed in this study is subject to the following licenses/restrictions: The original genotypes and cow phenotypes used for this study were supplied by CRV (https://www.crv4all-international.com/). The bull international breeding values (MACE) were available from Interbull (https://interbull.org/). Full details of the data and results supporting the discussions and conclusions are included in the article/Supplementary Material. Further inquiries regarding the raw data should be made to CRV. Requests to access these datasets should be directed to https://www.crv4all-international.com/ and https://interbull.org/.

## Ethics Statement

Ethical review and approval was not required for the animal study because no experimental animal procedures were conducted in this research.

## Author Contributions

HD, JP, IM, and CS conceived the study and developed the study design together with MG, MK, MH-M, and GJ. CS, GJ, EO’C, and EJ carried out the data pre-processing. MH-M and KK undertook the calculation of DRP. MK and IM performed the main data analysis. MK wrote the manuscript with assistance from IM. All the authors contributed to and approved the final manuscript.

## Conflict of Interest

KK and EJ were employed by DataGene. CS, GJ, and EO’C were employed by CRV, but they were not involved in the analysis of the data. The remaining authors declare that the research was conducted in the absence of any commercial or financial relationships that could be construed as a potential conflict of interest.

## Footnotes

1 https://www.dairynz.co.nz/publications/dairy-industry/new-zealand-dairy-statistics-2018-19/

2 https://datagene.com.au/

3 https://www.dairynz.co.nz/publications/dairy-industry/new-zealand-dairy-statistics-2018-19/

## Supplementary Material

Supplementary Figure 1 Accuracy and bias of genomic predictions in Refs. 1–5 (including Ref. 4’) using different marker sets (50k, XT_50k, and pruned HDnGBS) and analytic approaches (GBLUP and emBayesR) for milk, fat, and protein yields as well as averaged across milk traits.

Supplementary Figure 2 Accuracy and bias of genomic predictions in Ref. 3 and Refs. 6–8 using different marker sets (50k, XT_50k, and pruned HDnGBS) and analytic approaches (GBLUP and emBayesR) for milk, fat, and protein yields.
