# Supplementary material for: Improving Genomic Prediction of Crossbred and Purebred Dairy Cattle
Source: Front Genet. 2020 Dec 14;11:598580. doi: 10.3389/fgene.2020.598580 (PMC7767986; doi:10.3389/fgene.2020.598580)
Supplement: Supplementary Figure 1 — Accuracy and bias of genomic predictions in Refs. 1–5 (including Ref. 4’) using different marker sets (50k, XT_50k, and pruned HDnGBS) and analytic approaches (GBLUP and emBayesR) for milk, fat, and protein yields as well as averaged across milk traits. [file Data_Sheet_1.PDF]

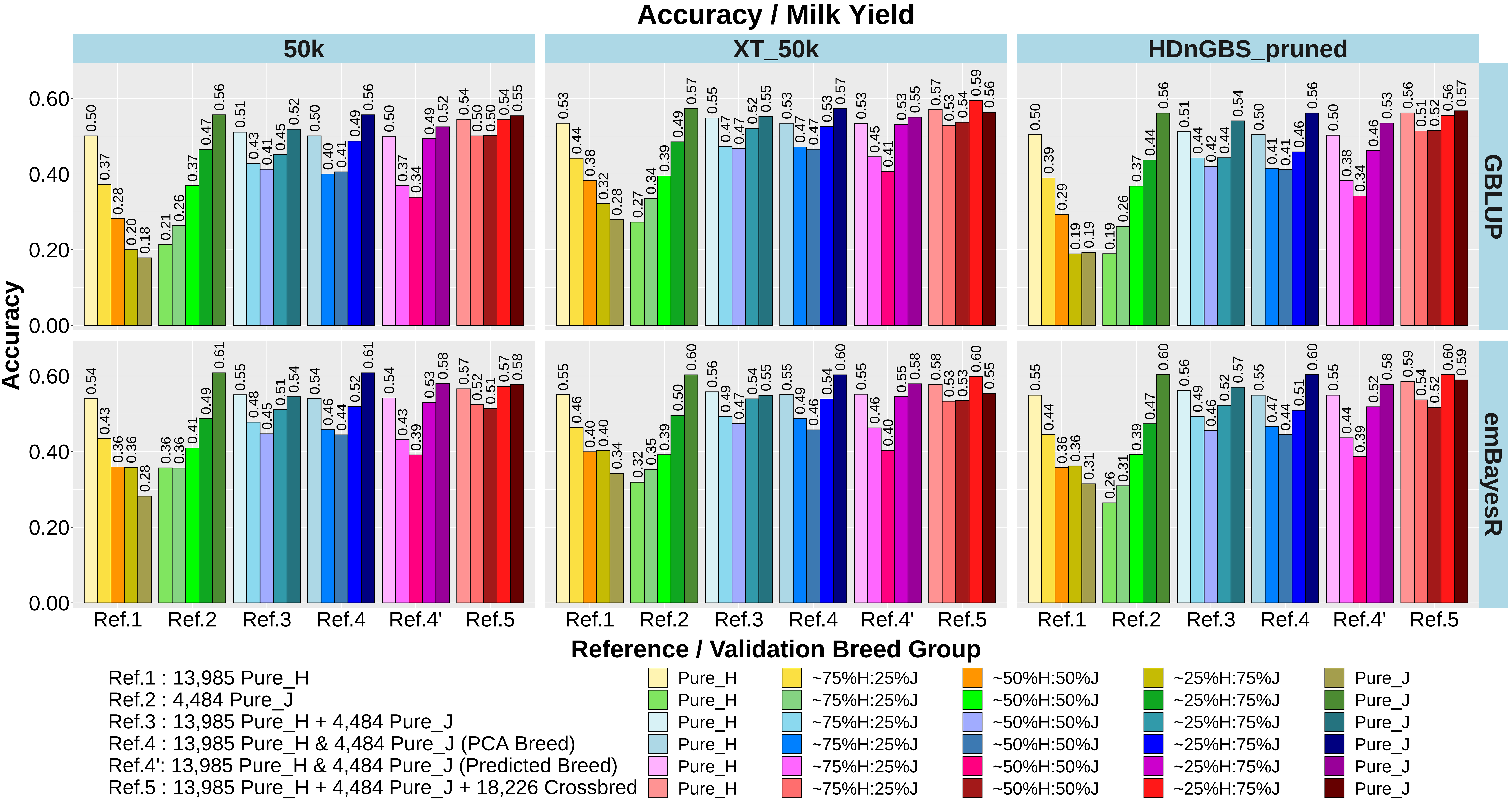

Accuracy / Milk Yield
50k
XT_50k
HDnGBS_pruned
GBLUP
emBayesR
Accuracy
0.00
0.20
0.40
0.60
0.50 0.37 0.28 0.20 0.18
0.21 0.26 0.37 0.47 0.56
0.51 0.43 0.41 0.45 0.52
0.50 0.40 0.41 0.49 0.56
0.50 0.37 0.34 0.49 0.52
0.54 0.50 0.50 0.54 0.55
0.53 0.44 0.38 0.32 0.28
0.27 0.34 0.39 0.49 0.57
0.55 0.47 0.47 0.52 0.55
0.53 0.47 0.47 0.53 0.57
0.53 0.45 0.41 0.53 0.55
0.57 0.53 0.54 0.59 0.56
0.50 0.39 0.29 0.19 0.19
0.19 0.26 0.37 0.44 0.56
0.51 0.44 0.42 0.44 0.54
0.50 0.41 0.41 0.46 0.56
0.50 0.38 0.34 0.46 0.53
0.56 0.51 0.52 0.56 0.57
0.54 0.43 0.36 0.36 0.28
0.36 0.36 0.41 0.49 0.61
0.55 0.48 0.45 0.51 0.54
0.54 0.46 0.44 0.52 0.61
0.54 0.43 0.39 0.53 0.58
0.57 0.52 0.51 0.57 0.58
0.55 0.46 0.40 0.40 0.34
0.32 0.35 0.39 0.50 0.60
0.56 0.49 0.47 0.54 0.55
0.55 0.49 0.46 0.54 0.60
0.55 0.46 0.40 0.55 0.58
0.58 0.53 0.53 0.60 0.55
0.55 0.44 0.36 0.36 0.31
0.26 0.31 0.39 0.47 0.60
0.56 0.49 0.46 0.52 0.57
0.55 0.47 0.44 0.51 0.60
0.55 0.44 0.39 0.52 0.58
0.59 0.54 0.52 0.60 0.59
Ref.1 Ref.2 Ref.3 Ref.4 Ref.4' Ref.5
Reference / Validation Breed Group
Ref.1 : 13,985 Pure_H
Ref.2 : 4,484 Pure_J
Ref.3 : 13,985 Pure_H + 4,484 Pure_J
Ref.4 : 13,985 Pure_H & 4,484 Pure_J (PCA Breed)
Ref.4': 13,985 Pure_H & 4,484 Pure_J (Predicted Breed)
Ref.5 : 13,985 Pure_H + 4,484 Pure_J + 18,226 Crossbred
Pure_H ~75%H:25%J ~50%H:50%J ~25%H:75%J Pure_J

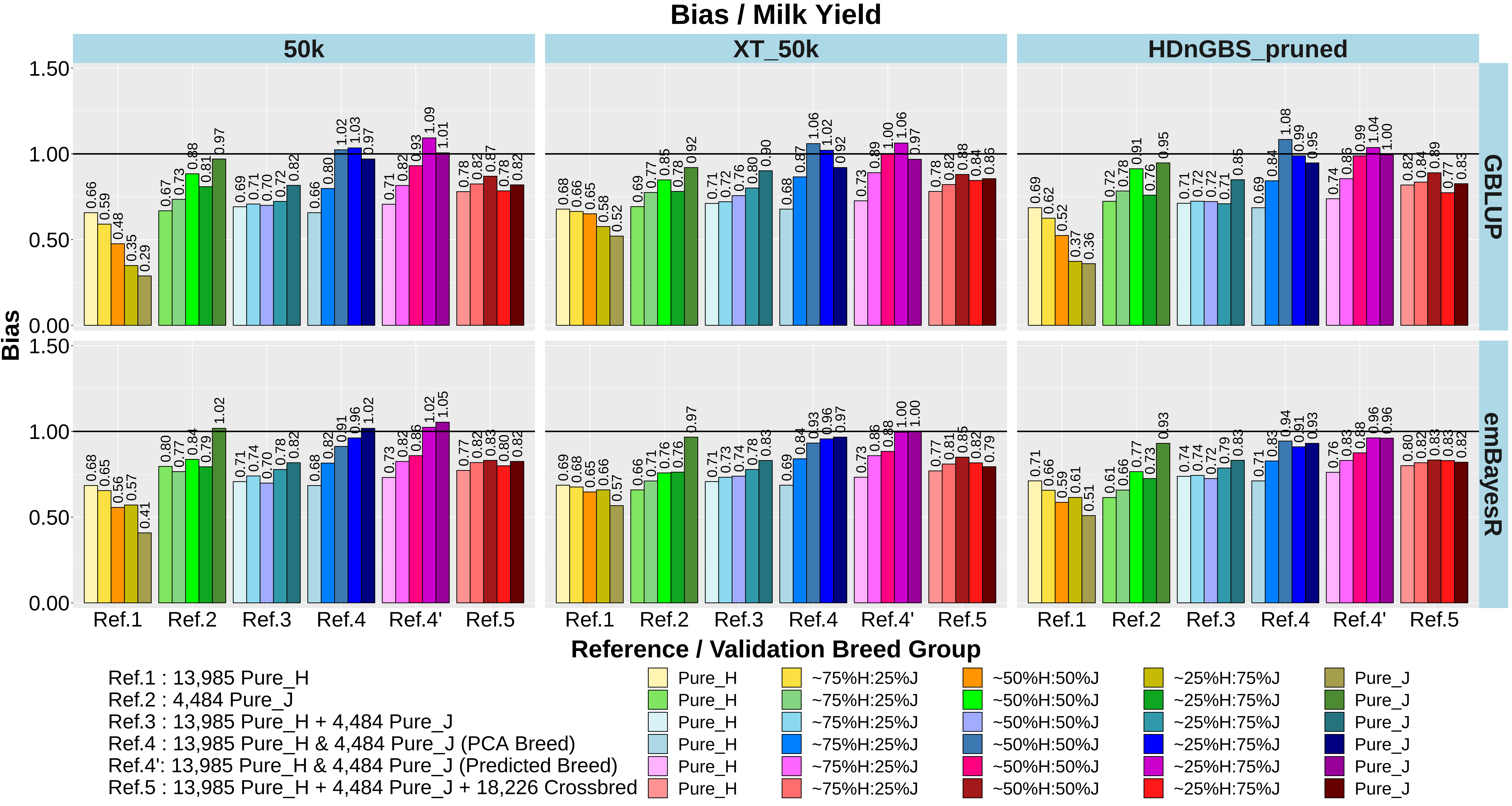
Bias / Milk Yield
50k
XT_50k
HDnGBS_pruned
GBLUP
emBayesR
Bias
1.50
1.00
0.50
0.00
0.66 0.59 0.48 0.35 0.29
0.67 0.73 0.88 0.81 0.97
0.69 0.71 0.70 0.72 0.82
0.66 0.80 1.02 1.03 0.97
0.71 0.82 0.93 1.09 1.01
0.78 0.82 0.87 0.78 0.82
0.68 0.66 0.65 0.58 0.52
0.69 0.77 0.85 0.78 0.92
0.71 0.72 0.76 0.80 0.90
0.68 0.87 1.06 1.02 0.92
0.73 0.89 1.00 1.06 0.97
0.78 0.82 0.88 0.84 0.86
0.69 0.62 0.52 0.37 0.36
0.72 0.78 0.91 0.76 0.95
0.71 0.72 0.72 0.71 0.85
0.69 0.84 1.08 0.99 0.95
0.74 0.86 0.99 1.04 1.00
0.82 0.84 0.89 0.77 0.83
0.68 0.65 0.56 0.57 0.41
0.80 0.77 0.84 0.79 1.02
0.71 0.74 0.70 0.78 0.82
0.68 0.82 0.91 0.96 1.02
0.73 0.82 0.86 1.02 1.05
0.77 0.82 0.83 0.80 0.82
0.69 0.68 0.65 0.66 0.57
0.66 0.71 0.76 0.76 0.97
0.71 0.73 0.74 0.78 0.83
0.69 0.84 0.93 0.96 0.97
0.73 0.86 0.88 1.00 1.00
0.77 0.81 0.85 0.82 0.79
0.71 0.66 0.59 0.61 0.51
0.61 0.66 0.77 0.73 0.93
0.74 0.74 0.72 0.79 0.83
0.71 0.83 0.94 0.91 0.93
0.76 0.83 0.88 0.96 0.96
0.80 0.82 0.83 0.83 0.82
Ref.1 Ref.2 Ref.3 Ref.4 Ref.4' Ref.5
Reference / Validation Breed Group
Ref.1 : 13,985 Pure_H
Ref.2 : 4,484 Pure_J
Ref.3 : 13,985 Pure_H + 4,484 Pure_J
Ref.4 : 13,985 Pure_H & 4,484 Pure_J (PCA Breed)
Ref.4': 13,985 Pure_H & 4,484 Pure_J (Predicted Breed)
Ref.5 : 13,985 Pure_H + 4,484 Pure_J + 18,226 Crossbred
Pure_H ~75%H:25%J ~50%H:50%J ~25%H:75%J Pure_J

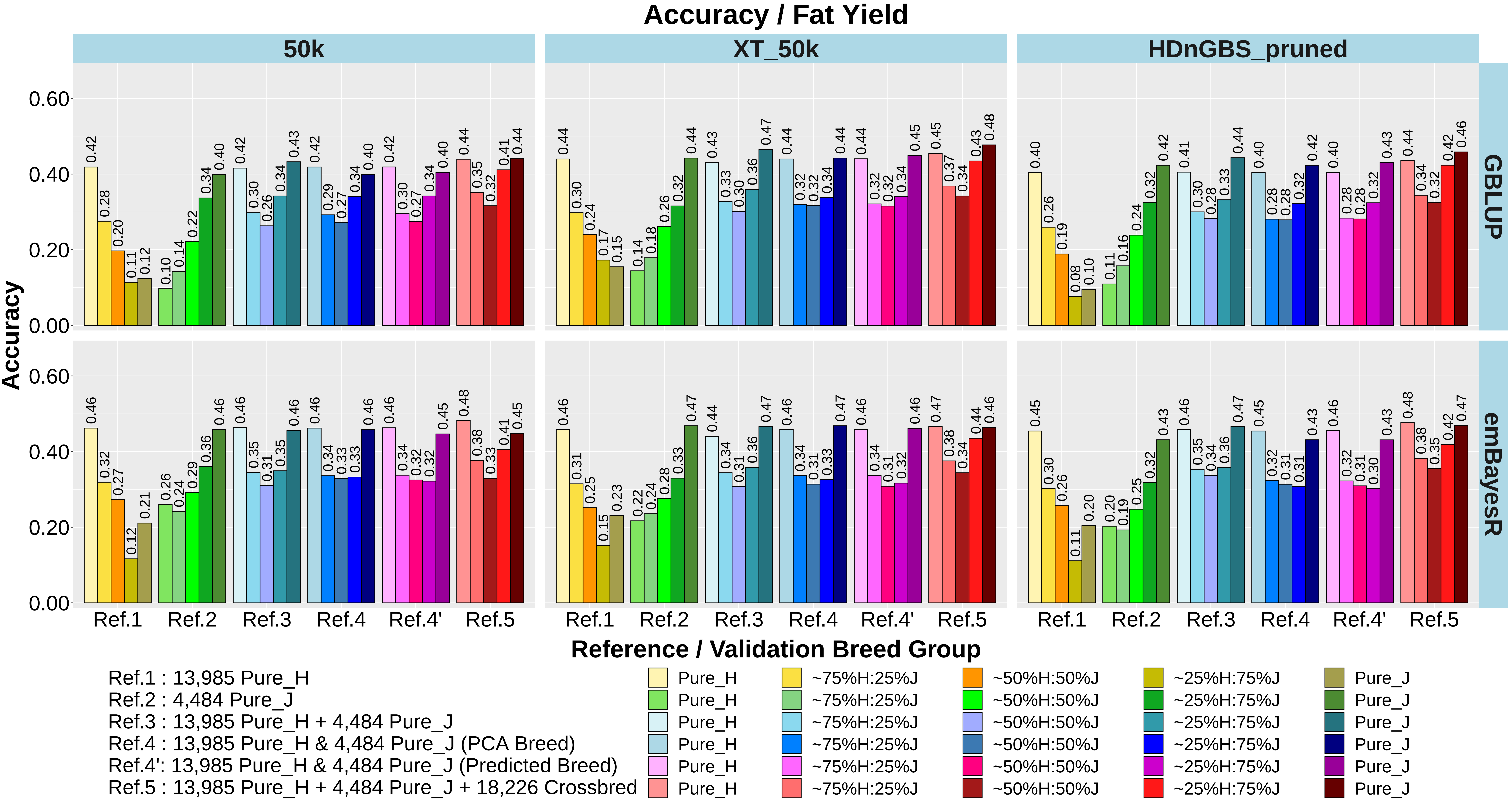
Accuracy / Fat Yield
50k
XT_50k
HDnGBS_pruned
GBLUP
emBayesR
Accuracy
Reference / Validation Breed Group
Ref.1
Ref.2
Ref.3
Ref.4
Ref.4'
Ref.5
Ref.1 : 13,985 Pure_H
Ref.2 : 4,484 Pure_J
Ref.3 : 13,985 Pure_H + 4,484 Pure_J
Ref.4 : 13,985 Pure_H & 4,484 Pure_J (PCA Breed)
Ref.4': 13,985 Pure_H & 4,484 Pure_J (Predicted Breed)
Ref.5 : 13,985 Pure_H + 4,484 Pure_J + 18,226 Crossbred
Pure_H
~75%H:25%J
~50%H:50%J
~25%H:75%J
Pure_J

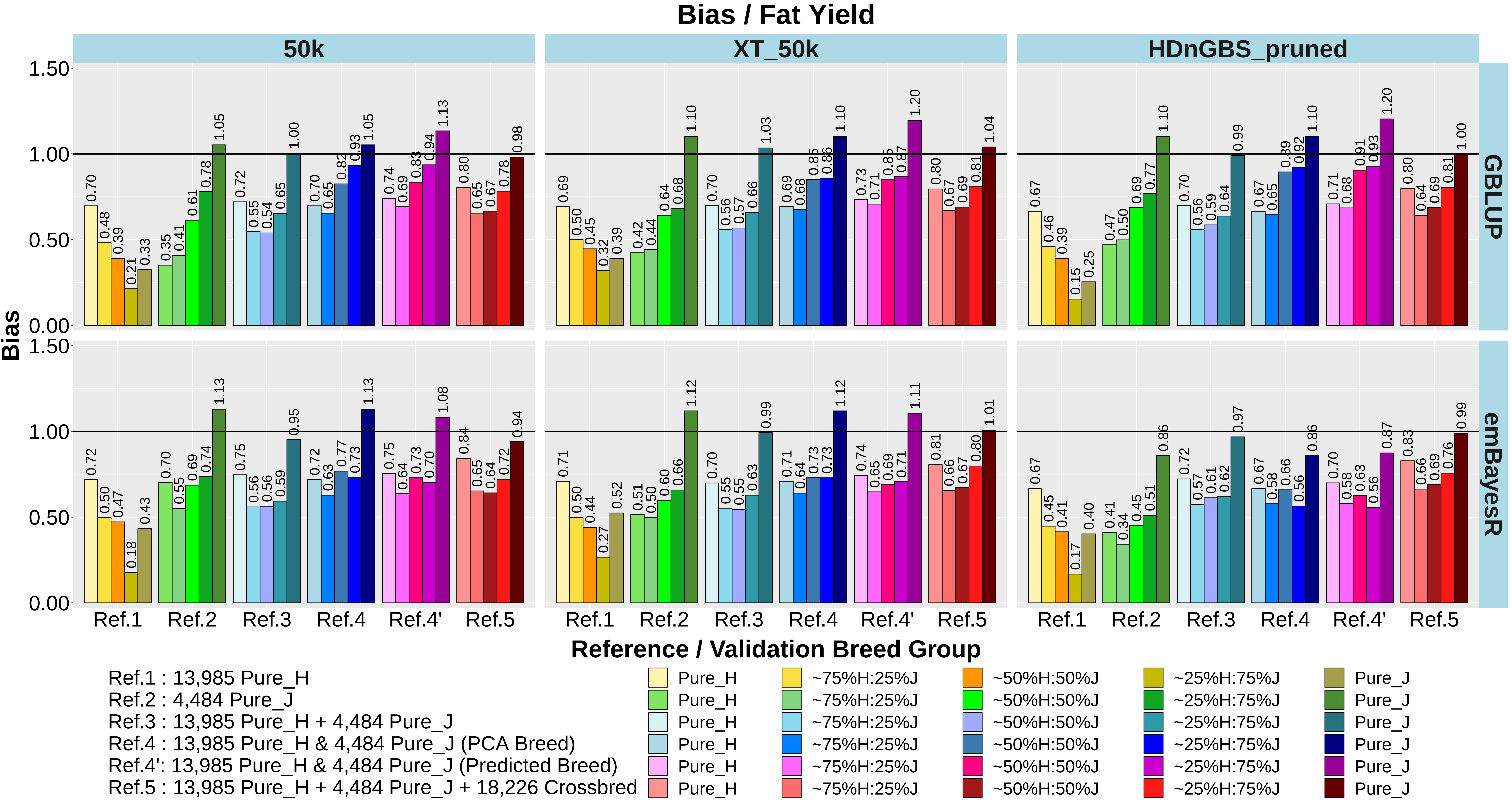
Bias / Fat Yield
50k
XT_50k
HDnGBS_pruned
GBLUP
emBayesR
Bias
1.50
1.00
0.50
0.00
Ref.1
Ref.2
Ref.3
Ref.4
Ref.4'
Ref.5
Reference / Validation Breed Group
Ref.1 : 13,985 Pure_H
Ref.2 : 4,484 Pure_J
Ref.3 : 13,985 Pure_H + 4,484 Pure_J
Ref.4 : 13,985 Pure_H & 4,484 Pure_J (PCA Breed)
Ref.4': 13,985 Pure_H & 4,484 Pure_J (Predicted Breed)
Ref.5 : 13,985 Pure_H + 4,484 Pure_J + 18,226 Crossbred
Pure_H
~75%H:25%J
~50%H:50%J
~25%H:75%J
Pure_J

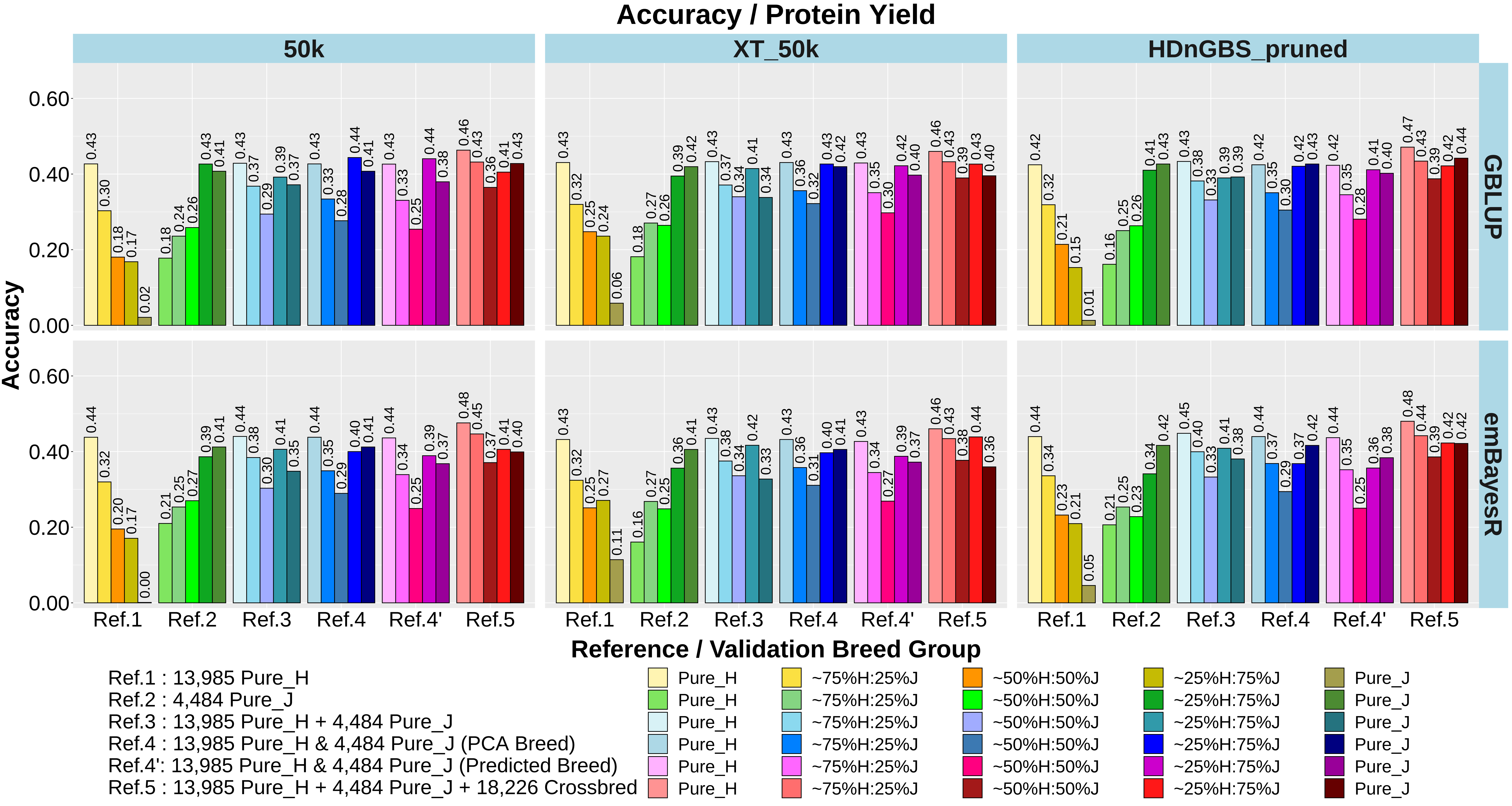

Accuracy / Protein Yield
50k
XT_50k
HDnGBS_pruned
GBLUP
emBayesR
Accuracy
0.00
0.20
0.40
0.60
0.43 0.30 0.18 0.17 0.02
0.18 0.24 0.26 0.43 0.41
0.43 0.37 0.29 0.39 0.37
0.43 0.33 0.28 0.44 0.41
0.43 0.33 0.25 0.44 0.38
0.46 0.43 0.36 0.41 0.43
0.43 0.32 0.25 0.24 0.06
0.18 0.27 0.26 0.39 0.42
0.43 0.37 0.34 0.41 0.34
0.43 0.36 0.32 0.43 0.42
0.43 0.35 0.30 0.42 0.40
0.46 0.43 0.39 0.43 0.40
0.42 0.32 0.21 0.15 0.01
0.16 0.25 0.26 0.41 0.43
0.43 0.38 0.33 0.39 0.39
0.42 0.35 0.30 0.42 0.43
0.42 0.35 0.28 0.41 0.40
0.47 0.43 0.39 0.42 0.44
0.44 0.32 0.20 0.17 0.00
0.21 0.25 0.27 0.39 0.41
0.44 0.38 0.30 0.41 0.35
0.44 0.35 0.29 0.40 0.41
0.44 0.34 0.25 0.39 0.37
0.48 0.45 0.37 0.41 0.40
0.43 0.32 0.25 0.27 0.11
0.16 0.27 0.25 0.36 0.41
0.43 0.38 0.34 0.42 0.33
0.43 0.36 0.31 0.40 0.41
0.43 0.34 0.27 0.39 0.37
0.46 0.43 0.38 0.44 0.36
0.44 0.34 0.23 0.21 0.05
0.21 0.25 0.23 0.34 0.42
0.45 0.40 0.33 0.41 0.38
0.44 0.37 0.29 0.37 0.42
0.44 0.35 0.25 0.36 0.38
0.48 0.44 0.39 0.42 0.42
Ref.1 Ref.2 Ref.3 Ref.4 Ref.4' Ref.5
Reference / Validation Breed Group
Ref.1 : 13,985 Pure_H
Ref.2 : 4,484 Pure_J
Ref.3 : 13,985 Pure_H + 4,484 Pure_J
Ref.4 : 13,985 Pure_H & 4,484 Pure_J (PCA Breed)
Ref.4': 13,985 Pure_H & 4,484 Pure_J (Predicted Breed)
Ref.5 : 13,985 Pure_H + 4,484 Pure_J + 18,226 Crossbred
Pure_H
~75%H:25%J
~50%H:50%J
~25%H:75%J
Pure_J

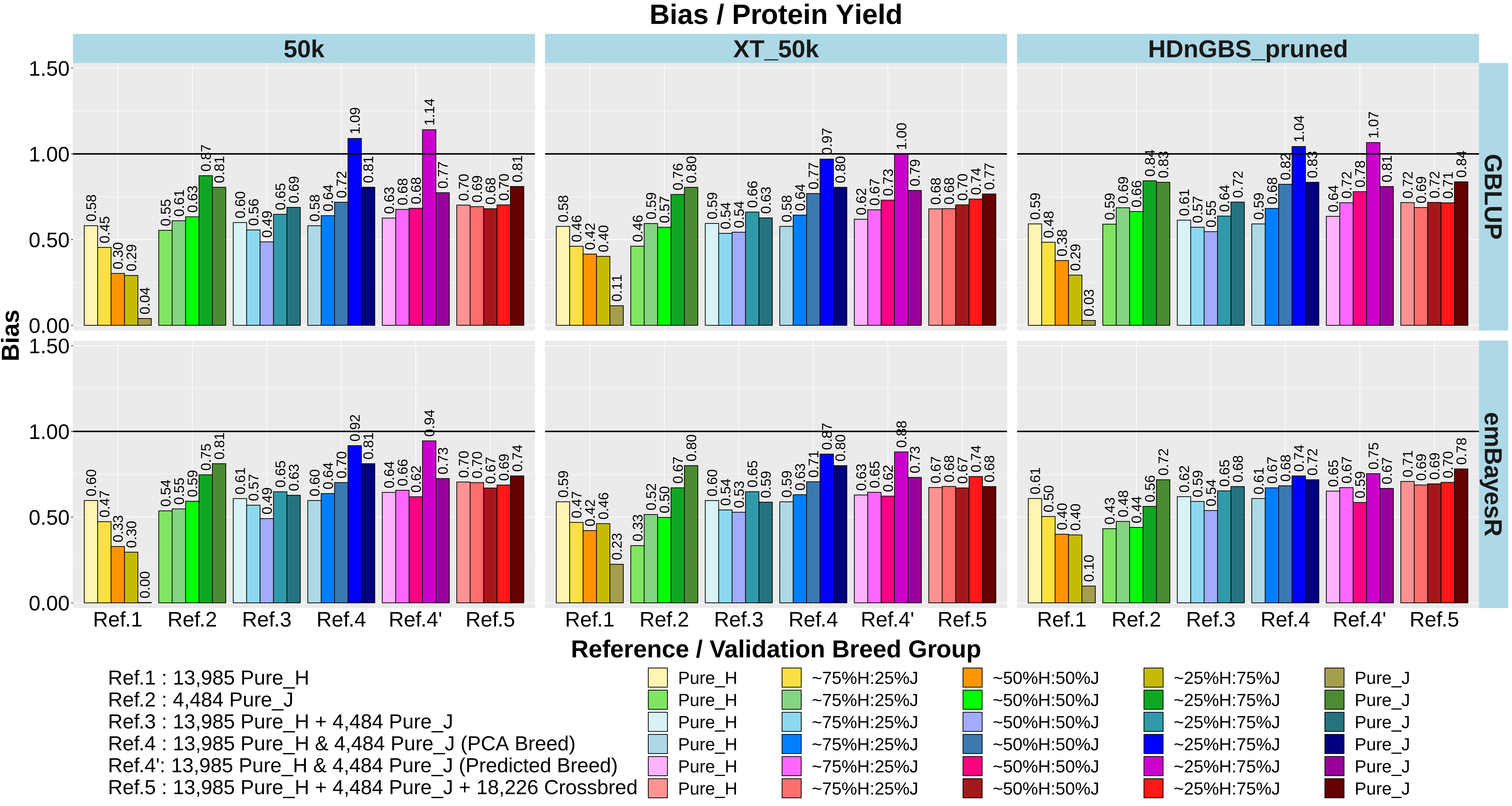
Bias / Protein Yield
50k
XT_50k
HDnGBS_pruned
GBLUP
emBayesR
Bias
1.50
1.00
0.50
0.00
Ref.1
Ref.2
Ref.3
Ref.4
Ref.4'
Ref.5
Reference / Validation Breed Group
Ref.1 : 13,985 Pure_H
Ref.2 : 4,484 Pure_J
Ref.3 : 13,985 Pure_H + 4,484 Pure_J
Ref.4 : 13,985 Pure_H & 4,484 Pure_J (PCA Breed)
Ref.4': 13,985 Pure_H & 4,484 Pure_J (Predicted Breed)
Ref.5 : 13,985 Pure_H + 4,484 Pure_J + 18,226 Crossbred
Pure_H
~75%H:25%J
~50%H:50%J
~25%H:75%J
Pure_J

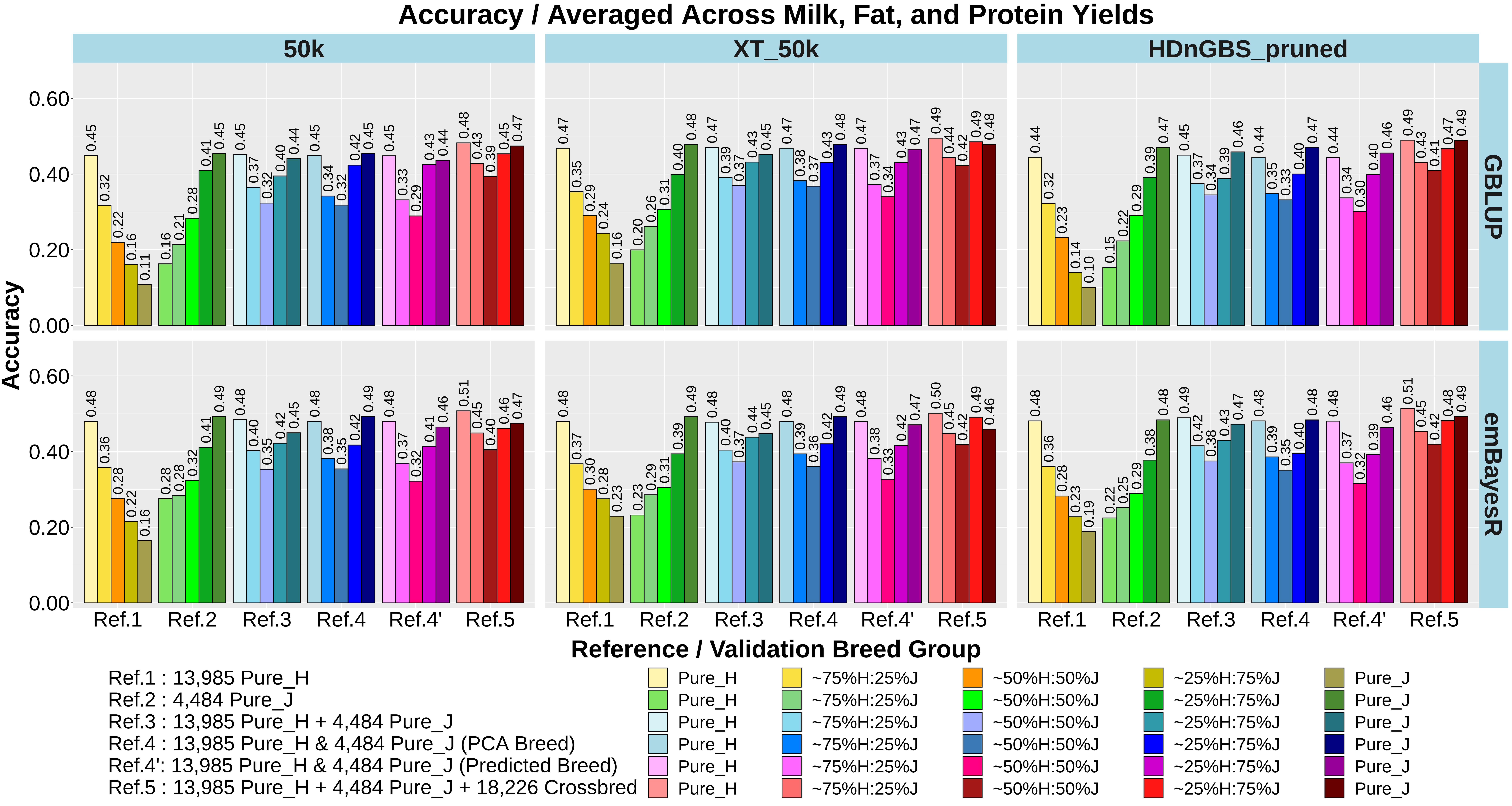
Accuracy / Averaged Across Milk, Fat, and Protein Yields
50k
XT_50k
HDnGBS_pruned
GBLUP
emBayesR
Accuracy
0.00
0.20
0.40
0.60
0.45 0.32 0.22 0.16 0.11
0.16 0.21 0.28 0.41 0.45
0.45 0.37 0.32 0.40 0.44
0.45 0.34 0.32 0.42 0.45
0.45 0.33 0.29 0.43 0.44
0.48 0.43 0.39 0.45 0.47
0.47 0.35 0.29 0.24 0.16
0.20 0.26 0.31 0.40 0.48
0.47 0.39 0.37 0.43 0.45
0.47 0.38 0.37 0.43 0.48
0.47 0.37 0.34 0.43 0.47
0.49 0.44 0.42 0.49 0.48
0.44 0.32 0.23 0.14 0.10
0.15 0.22 0.29 0.39 0.47
0.45 0.37 0.34 0.39 0.46
0.44 0.35 0.33 0.40 0.47
0.44 0.34 0.30 0.40 0.46
0.49 0.43 0.41 0.47 0.49
0.48 0.36 0.28 0.22 0.16
0.28 0.28 0.32 0.41 0.49
0.48 0.40 0.35 0.42 0.45
0.48 0.38 0.35 0.42 0.49
0.48 0.37 0.32 0.41 0.46
0.51 0.45 0.40 0.46 0.47
0.48 0.37 0.30 0.28 0.23
0.23 0.29 0.31 0.39 0.49
0.48 0.40 0.37 0.44 0.45
0.48 0.39 0.36 0.42 0.49
0.48 0.38 0.33 0.42 0.47
0.50 0.45 0.42 0.49 0.46
0.48 0.36 0.28 0.23 0.19
0.22 0.25 0.29 0.38 0.48
0.49 0.42 0.38 0.43 0.47
0.48 0.39 0.35 0.40 0.48
0.48 0.37 0.32 0.39 0.46
0.51 0.45 0.42 0.48 0.49
Ref.1 Ref.2 Ref.3 Ref.4 Ref.4' Ref.5
Reference / Validation Breed Group
Ref.1 : 13,985 Pure_H
Ref.2 : 4,484 Pure_J
Ref.3 : 13,985 Pure_H + 4,484 Pure_J
Ref.4 : 13,985 Pure_H & 4,484 Pure_J (PCA Breed)
Ref.4': 13,985 Pure_H & 4,484 Pure_J (Predicted Breed)
Ref.5 : 13,985 Pure_H + 4,484 Pure_J + 18,226 Crossbred
Pure_H ~75%H:25%J ~50%H:50%J ~25%H:75%J Pure_J

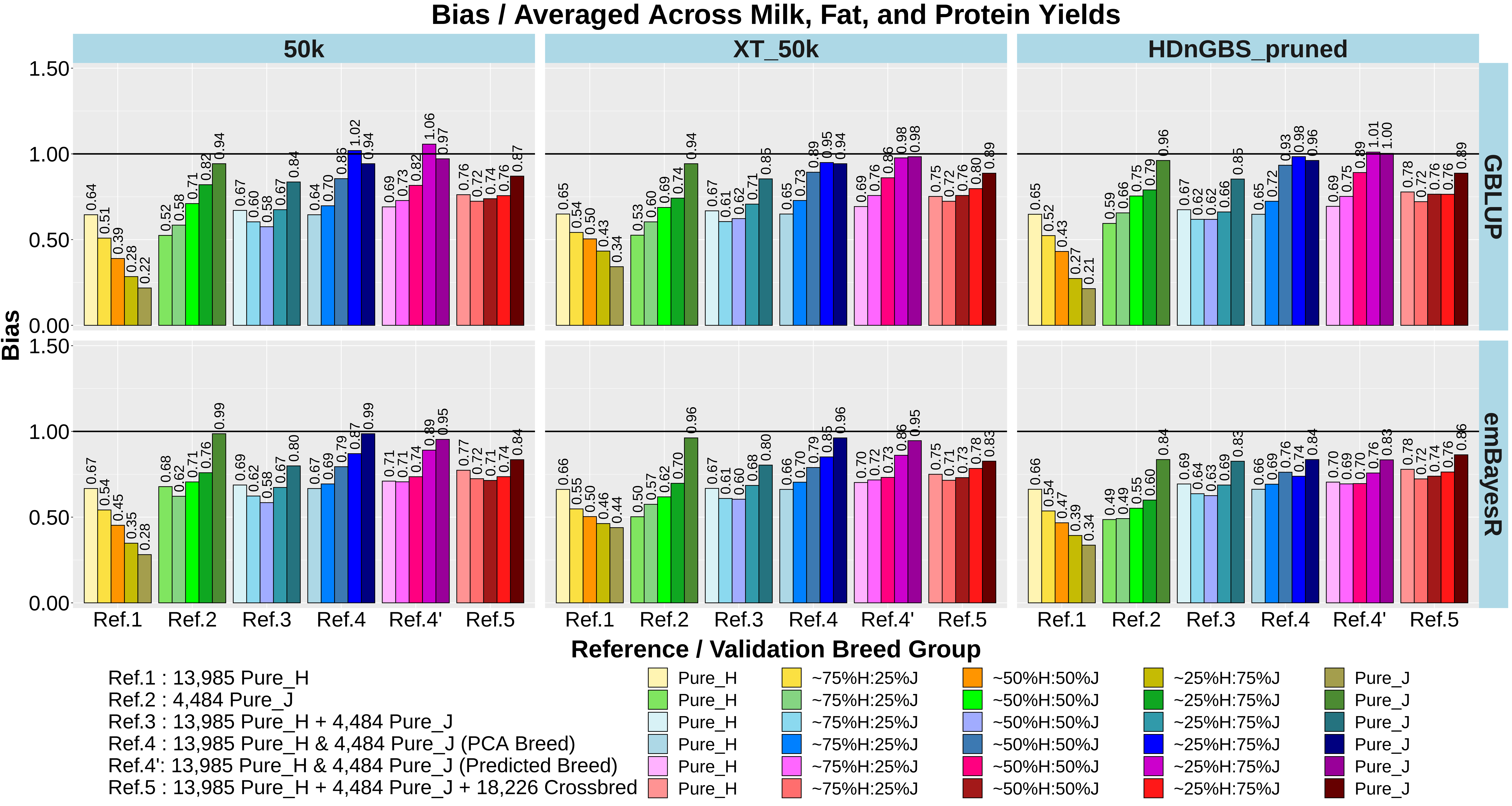
Bias / Averaged Across Milk, Fat, and Protein Yields
50k
XT_50k
HDnGBS_pruned
GBLUP
emBayesR
Bias
1.50
1.00
0.50
0.00
Ref.1
Ref.2
Ref.3
Ref.4
Ref.4'
Ref.5
Reference / Validation Breed Group
Ref.1 : 13,985 Pure_H
Ref.2 : 4,484 Pure_J
Ref.3 : 13,985 Pure_H + 4,484 Pure_J
Ref.4 : 13,985 Pure_H & 4,484 Pure_J (PCA Breed)
Ref.4': 13,985 Pure_H & 4,484 Pure_J (Predicted Breed)
Ref.5 : 13,985 Pure_H + 4,484 Pure_J + 18,226 Crossbred
Pure_H
~75%H:25%J
~50%H:50%J
~25%H:75%J
Pure_J
