# Supplementary material for: Improving Genomic Prediction of Crossbred and Purebred Dairy Cattle
Source: Front Genet. 2020 Dec 14;11:598580. doi: 10.3389/fgene.2020.598580 (PMC7767986; doi:10.3389/fgene.2020.598580)
Supplement: Supplementary Figure 2 — Accuracy and bias of genomic predictions in Ref. 3 and Refs. 6–8 using different marker sets (50k, XT_50k, and pruned HDnGBS) and analytic approaches (GBLUP and emBayesR) for milk, fat, and protein yields. [file Data_Sheet_2.PDF]

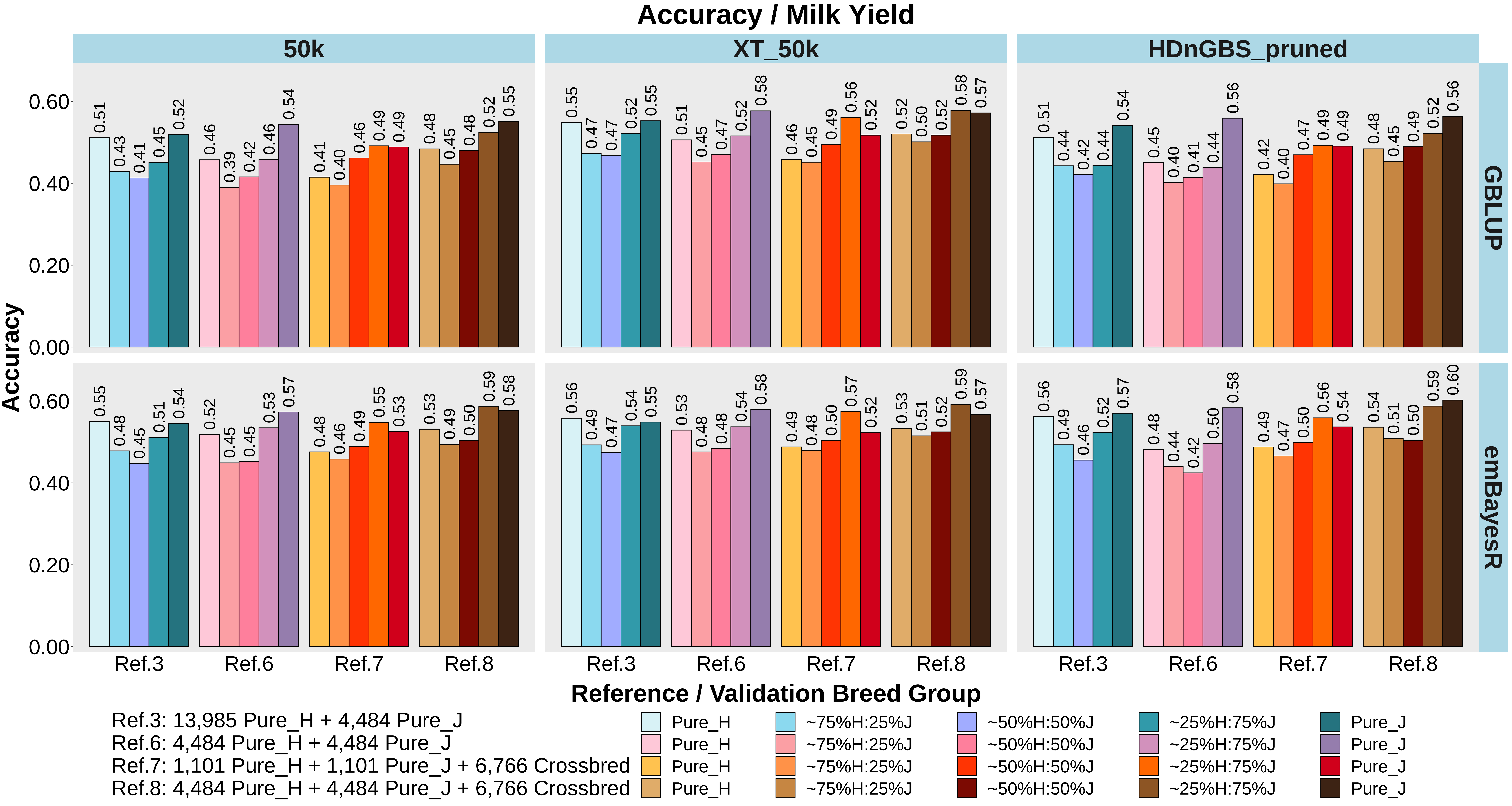
Accuracy / Milk Yield
50k
XT_50k
HDnGBS_pruned
GBLUP
emBayesR
Accuracy
0.00
0.20
0.40
0.60
Ref.3
Ref.6
Ref.7
Ref.8
Reference / Validation Breed Group
Ref.3: 13,985 Pure_H + 4,484 Pure_J
Ref.6: 4,484 Pure_H + 4,484 Pure_J
Ref.7: 1,101 Pure_H + 1,101 Pure_J + 6,766 Crossbred
Ref.8: 4,484 Pure_H + 4,484 Pure_J + 6,766 Crossbred
Pure_H
~75%H:25%J
~50%H:50%J
~25%H:75%J
Pure_J

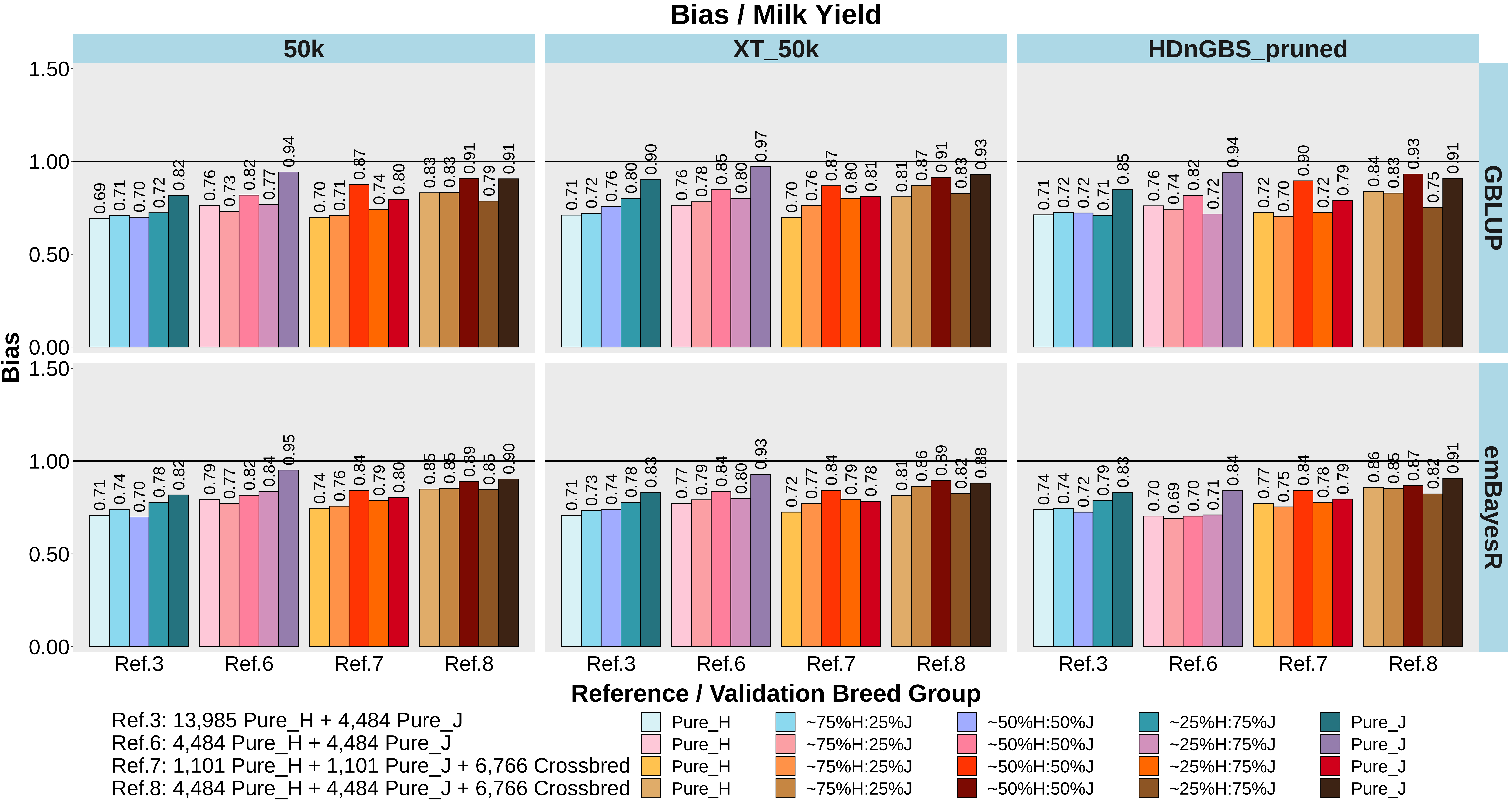

Bias / Milk Yield
50k
XT_50k
HDnGBS_pruned
GBLUP
emBayesR
Bias
1.50
1.00
0.50
0.00
0.69 0.71 0.70 0.72 0.82
0.76 0.73 0.82 0.77 0.94
0.70 0.71 0.87 0.74 0.80
0.83 0.83 0.91 0.79 0.91
0.71 0.72 0.76 0.80 0.90
0.76 0.78 0.85 0.80 0.97
0.70 0.76 0.87 0.80 0.81
0.81 0.87 0.91 0.83 0.93
0.71 0.72 0.72 0.71 0.85
0.76 0.74 0.82 0.72 0.94
0.72 0.70 0.90 0.72 0.79
0.84 0.83 0.93 0.75 0.91
0.71 0.74 0.70 0.78 0.82
0.79 0.77 0.82 0.84 0.95
0.74 0.76 0.84 0.79 0.80
0.85 0.85 0.89 0.85 0.90
0.71 0.73 0.74 0.78 0.83
0.77 0.79 0.84 0.80 0.93
0.72 0.77 0.84 0.79 0.78
0.81 0.86 0.89 0.82 0.88
0.74 0.74 0.72 0.79 0.83
0.70 0.69 0.70 0.71 0.84
0.77 0.75 0.84 0.78 0.79
0.86 0.85 0.87 0.82 0.91
Ref.3
Ref.6
Ref.7
Ref.8
Reference / Validation Breed Group
Ref.3: 13,985 Pure_H + 4,484 Pure_J
Ref.6: 4,484 Pure_H + 4,484 Pure_J
Ref.7: 1,101 Pure_H + 1,101 Pure_J + 6,766 Crossbred
Ref.8: 4,484 Pure_H + 4,484 Pure_J + 6,766 Crossbred
Pure_H
~75%H:25%J
~50%H:50%J
~25%H:75%J
Pure_J

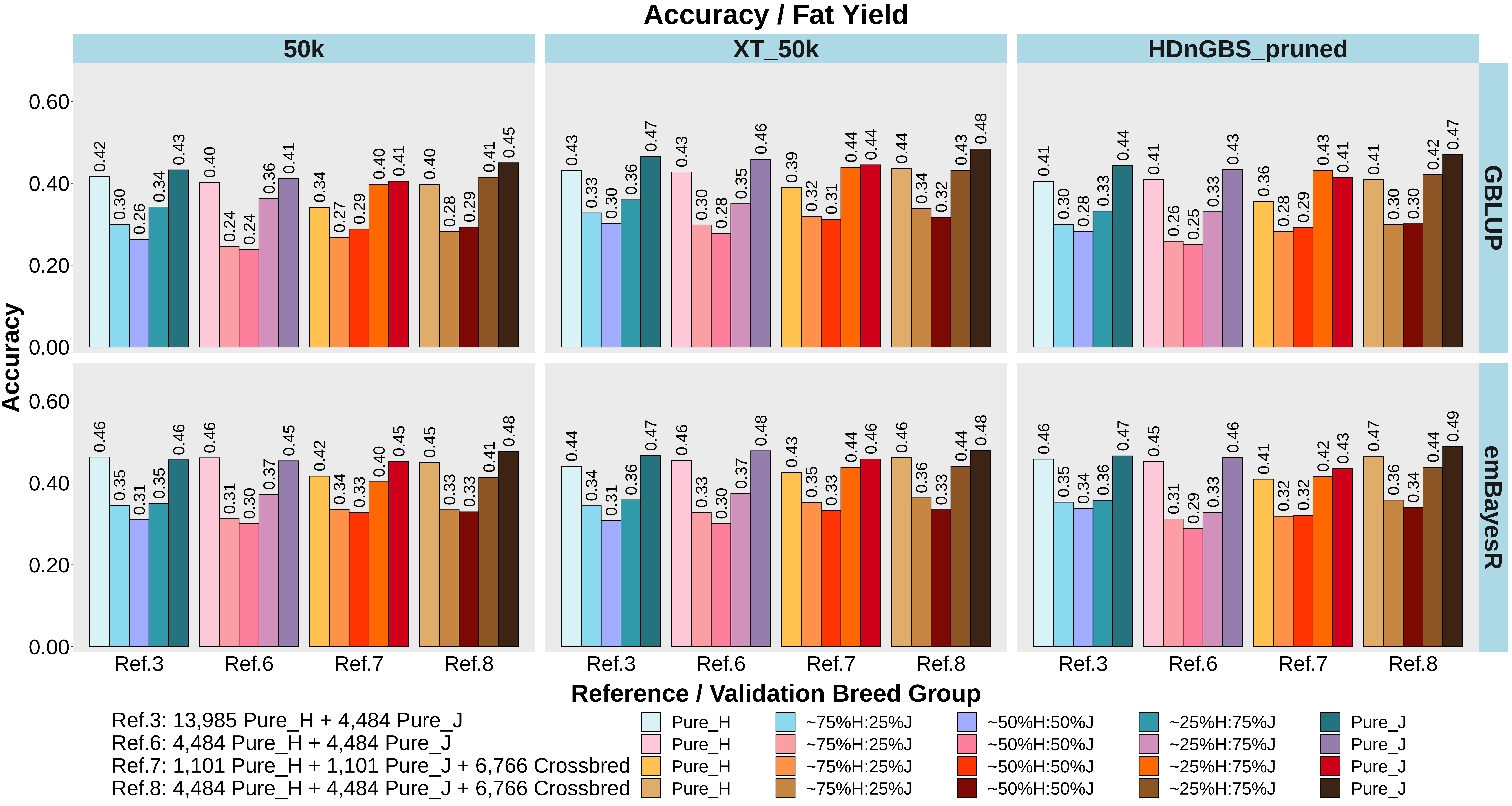
Accuracy / Fat Yield
50k
XT_50k
HDnGBS_pruned
GBLUP
emBayesR
Accuracy
0.00
0.20
0.40
0.60
Ref.3
Ref.6
Ref.7
Ref.8
Reference / Validation Breed Group
Ref.3: 13,985 Pure_H + 4,484 Pure_J
Ref.6: 4,484 Pure_H + 4,484 Pure_J
Ref.7: 1,101 Pure_H + 1,101 Pure_J + 6,766 Crossbred
Ref.8: 4,484 Pure_H + 4,484 Pure_J + 6,766 Crossbred
Pure_H
~75%H:25%J
~50%H:50%J
~25%H:75%J
Pure_J

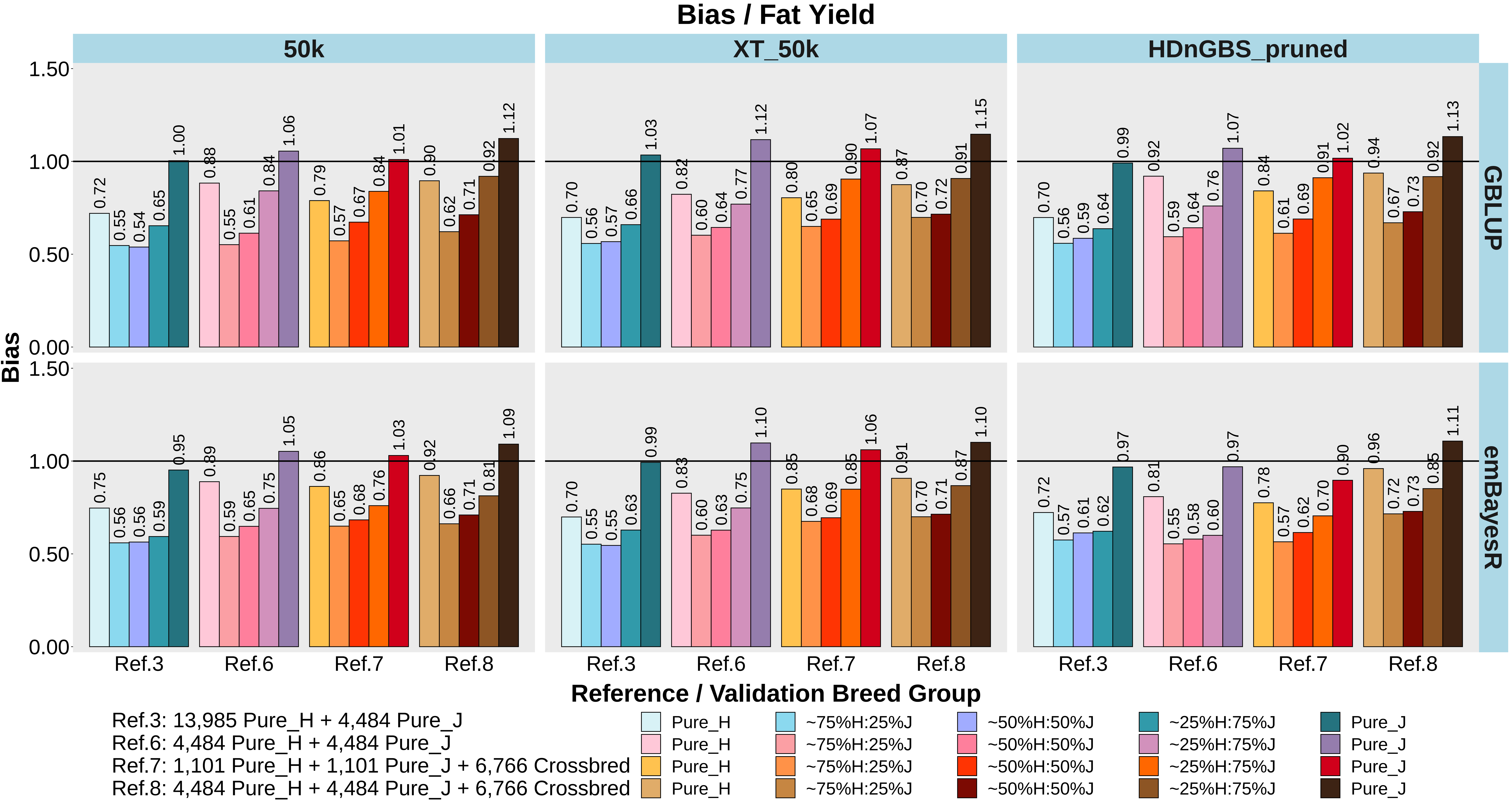

Bias / Fat Yield
50k
XT_50k
HDnGBS_pruned
GBLUP
emBayesR
Bias
Reference / Validation Breed Group
Ref.3
Ref.6
Ref.7
Ref.8
Ref.3: 13,985 Pure_H + 4,484 Pure_J
Ref.6: 4,484 Pure_H + 4,484 Pure_J
Ref.7: 1,101 Pure_H + 1,101 Pure_J + 6,766 Crossbred
Ref.8: 4,484 Pure_H + 4,484 Pure_J + 6,766 Crossbred
Pure_H
~75%H:25%J
~50%H:50%J
~25%H:75%J
Pure_J

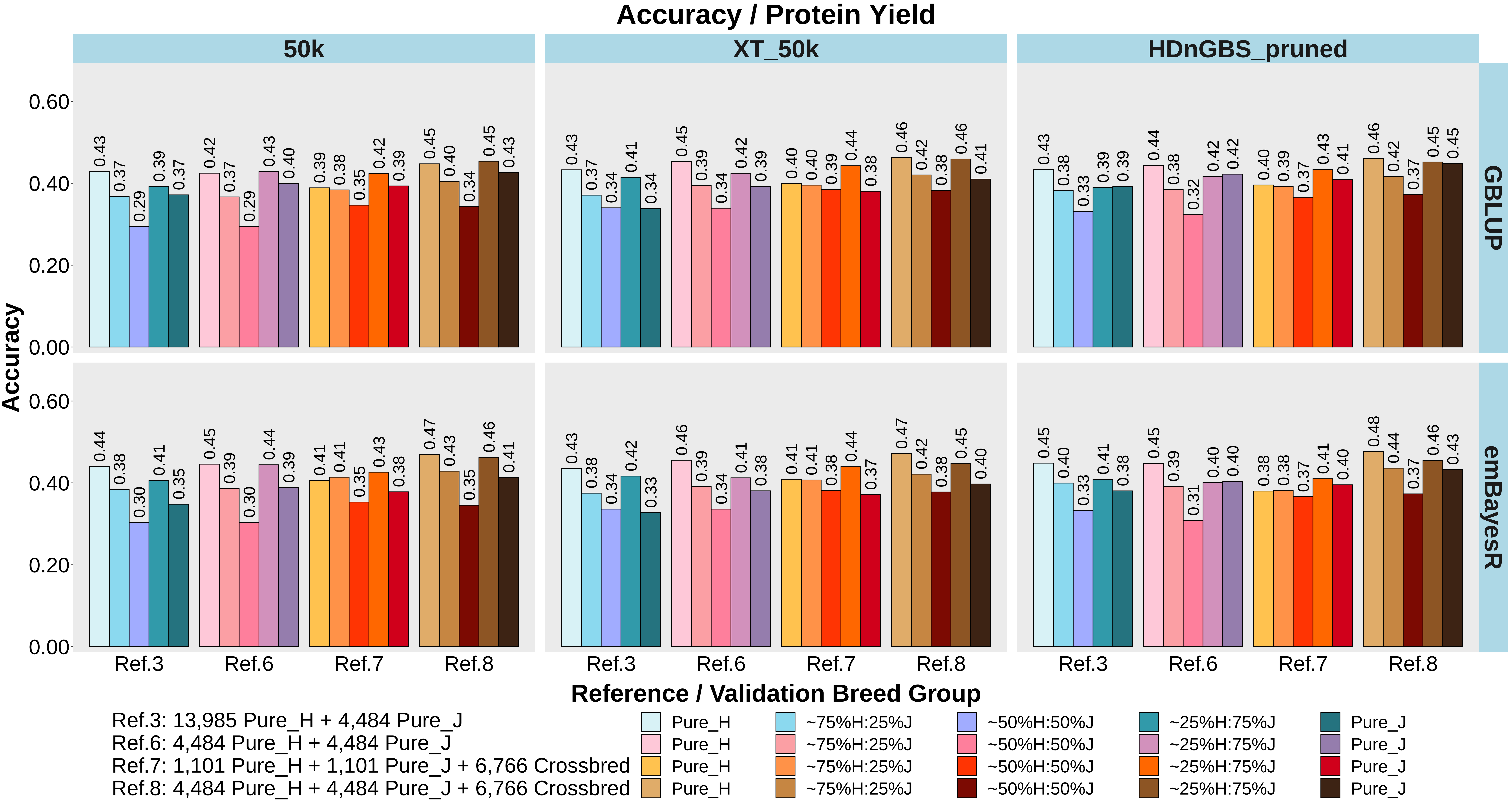

Accuracy / Protein Yield
50k
XT_50k
HDnGBS_pruned
GBLUP
emBayesR
Accuracy
0.00
0.20
0.40
0.60
Ref.3
Ref.6
Ref.7
Ref.8
Reference / Validation Breed Group
Ref.3: 13,985 Pure_H + 4,484 Pure_J
Ref.6: 4,484 Pure_H + 4,484 Pure_J
Ref.7: 1,101 Pure_H + 1,101 Pure_J + 6,766 Crossbred
Ref.8: 4,484 Pure_H + 4,484 Pure_J + 6,766 Crossbred
Pure_H
~75%H:25%J
~50%H:50%J
~25%H:75%J
Pure_J

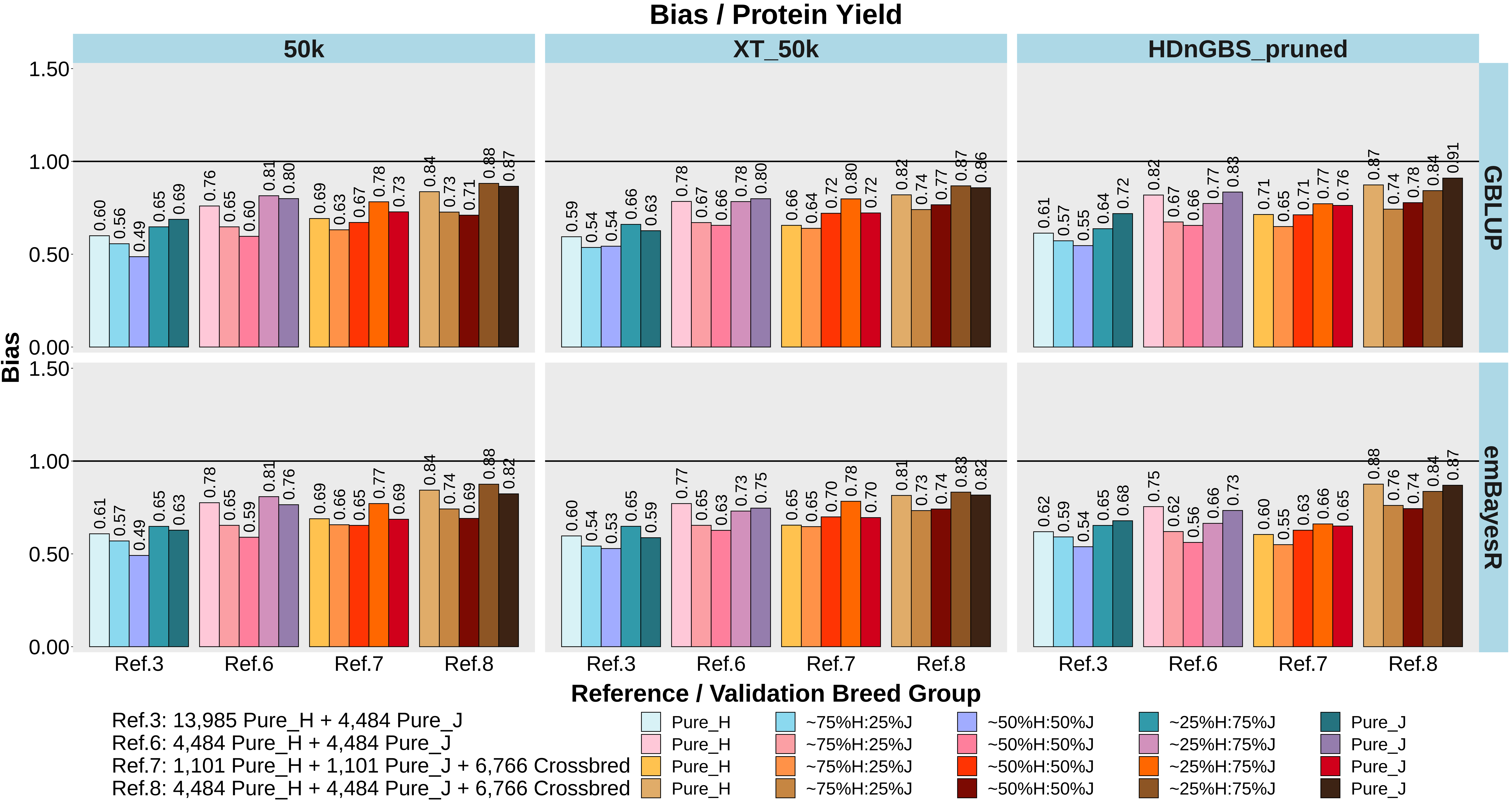
Bias / Protein Yield
50k
XT_50k
HDnGBS_pruned
GBLUP
emBayesR
Bias
Reference / Validation Breed Group
Ref.3
Ref.6
Ref.7
Ref.8
Ref.3: 13,985 Pure_H + 4,484 Pure_J
Ref.6: 4,484 Pure_H + 4,484 Pure_J
Ref.7: 1,101 Pure_H + 1,101 Pure_J + 6,766 Crossbred
Ref.8: 4,484 Pure_H + 4,484 Pure_J + 6,766 Crossbred
Pure_H
~75%H:25%J
~50%H:50%J
~25%H:75%J
Pure_J
